# Health assessments and screening tools for adults experiencing homelessness: a systematic review

**DOI:** 10.1186/s12889-019-7234-y

**Published:** 2019-07-24

**Authors:** S. J. Gordon, K. Grimmer, A. Bradley, T. Direen, N. Baker, T. Marin, M. T. Kelly, S. Gardner, M. Steffens, T. Burgess, C. Hume, J. L. Oliffe

**Affiliations:** 1 College of Nursing and Health Sciences, Flinders University, Bedford Park, South Australia 5042; 2 Division of Physiotherapy, Faculty of Medicine and Health Science, Stellenbosch Uni, Cape Town, South Africa; 3 Adelaide Dental School, University of Adelaide, Adelaide, South Australia 5000; 4 School of Public Health, University of Adelaide, Adelaide, South Australia 5000; 5 School of Nursing, University of British Columbia, Vancouver, Canada

**Keywords:** Homeless adults, Health assessment, Screening, Review, Homelessness

## Abstract

**Background:**

Homelessness is increasing globally. It results in poorer physical and mental health than age matched people living in permanent housing. Better information on the health needs of people experiencing homelessness is needed to inform effective resourcing, planning and service delivery by government and care organisations.

The aim of this review was to identify assessment tools that are valid, reliable and appropriate to measure the health status of people who are homeless.

**Methods:**

Data sources: A systematic literature search was conducted in PubMed (and Medline), PsychInfo, Scopus, CINAHL and ERIC from database inception until September 2018. Key words used were homeless, homelessness, homeless persons, vagrancy, health status, health, health issues, health assessment and health screening. The protocol was registered with PROSPERO. The National Health and Medical Research Council of Australia (NHMRC) hierarchy of evidence was applied; methodological quality of included articles was assessed using the McMaster critical appraisal tools and psychometric properties of the tools were appraised using the International Centre for Allied Health Evidence Ready Reckoner.

**Results:**

Diverse tools and measures (*N* = 71) were administered within, and across the reviewed studies (*N* = 37), with the main focus being on general health, oral health and nutrition. Eleven assessment tools in 13 studies had evidence of appropriate psychometric testing for the target population in domains of quality of life and health status, injury, substance use, mental health, psychological and cognitive function. Methodological quality of articles and tools were assessed as moderate to good. No validated tools were identified to assess oral health, chronic conditions, anthropometry, demography, nutrition, continence, functional decline and frailty, or vision and hearing. However, assessments of physical constructs (such as oral health, anthropometry, vision and hearing) could be applied to homeless people on a presumption of validity, because the constructs would be measured with clinical indicators in the same manner as people living in permanent dwellings.

**Conclusions:**

This review highlighted the need to develop consistent and comprehensive health assessment tools validated with, and tailored for, adults experiencing homelessness.

**Electronic supplementary material:**

The online version of this article (10.1186/s12889-019-7234-y) contains supplementary material, which is available to authorized users.

## Background

Homelessness is a serious global problem [1] that affects adults of all ages [2]. National assessments of homelessness vary in the definition of homelessness that is applied, making direct comparisons between countries difficult. In the European Union, it is estimated that 4.1 million people have a homeless episode in a year [3] while, based on shelter use, an estimated 150,000–300,000 people experience homelessness in Canada in a year [4].

The definitions of homelessness from the Australian Bureau of Statistics’ (ABS) [5] used in this review were*;*

- *a person living in streets or without a shelter that would fall within the scope of living quarters, [if a person has] no place of usual residence who move frequently between various types of accommodation (including dwellings, shelters or other living quarters) or [if a person is] usually resident in long-term shelters or similar arrangements for the homeles (*pp. 5*);* OR
- *a person living “in a dwelling that is inadequate; or has no tenure, or if their initial tenure is short and not extendable; or does not allow them to have control of, and access to space for social relations”* (pp. 11), or

These are broad definitions that include people experiencing homelessness in different ways, such as a sofa surfer or someone with insecure housing tenure. The definitions thus reflect the variability and changing circumstances of people who experience homelessness. Applying this definition, the Australian census found 116,000 people were homeless on Census night in 2016, representing 50 homeless people per 10,000 [5].

The recent Rough Sleeping Statistics (Autumn 2017) for England [6] counts:

- People sleeping, about to bed down (sitting on/in or standing next to their bedding) or actually bedded down in the open air (such as on the streets, in tents, doorways, parks, bus shelters or encampments), and
- People in buildings or other places not designed for habitation (such as stairwells, barns, sheds, car parks, cars, derelict boats, stations, or “bashes” which are makeshift shelters, often comprised of cardboard boxes).

These definitions do not include people in hostels or shelters, people in campsites or other sites used for recreational purposes or organised protest, squatters or travellers [6]. This may explain why the single night snapshot for England was 4751 people or 20 per 10,000 [6], was so much lower than Australian Figs. [5]. What is concerning is that irrespective of the definition used, the rate of homelessness in Australia had increased by 4.6% between 2011 and 2016 [5], whilst in England there was an 18% increase in London and a 14% increase in the rest of England between 2016 and 2017, of people sleeping rough [6].

The state of being homeless impacts significantly on health [7], resulting in higher rates of premature mortality [4], with the average age of death for men and women experiencing homelessness in England being 47 and 43 years respectively [7]. In Canada and the USA, the current mean age of people experiencing homelessness is approximately 50 years [8–10].

Consequently, many people experiencing homelessness also present with age-related declining physical and cognitive functions [4, 9, 10]. Compared to the general population, people experiencing homelessness have higher levels of mental illness [8], drug and alcohol use [4], infectious diseases, including influenza, tuberculosis, human immunodeficiency virus (HIV), hepatitis, and sexually transmitted infections [11], oral cancer and other dental problems [7, 8], injury and assault and skin problems (often related to cold exposure) [11]. Approximately 40% adults experiencing homelessness report having at least one chronic health condition, which is often poorly controlled [7, 12]. Compounding poor health is the transient nature of homelessness, which often precludes sustained management of these health conditions [7]. For instance, people experiencing homelessness typically attend acute care services, such as hospital emergency departments, when they experience health crises, rather than accessing planned, preventative models of care [13].

Appropriate assessment tools are needed to identify the health needs of adults experiencing homelessness. Appropriate assessments can inform the development of policies and practices to provide effective prevention of health problems, and interventions that improve health. Appropriate in this context means that the assessment should use language, items, and constructs which are relevant to homelessness. For instance, many validated tools are available to evaluate sleep quality, nutrition and hygiene in the general population. These tools often have questions worded in a manner that assume that respondents sleep in a bed, and have access to food preparation and storage services, as well as bathroom and toilet facilities. As this is may not be the case for many people experiencing homelessness, it is important that health screening tools are properly validated and appropriate for specific circumstances of homelessness.

The usefulness of health screening and assessment tools to collect relevant information on particular individuals, and/or a specific target population is typically expressed in terms of validity, reliability and utility of application. Testing for validity establishes that the health screening or assessment tool captures all the constructs (or elements) that it purports to measure [14], and that the scores compare with other similar measures [14]. Reliability is about understanding variability and error in repeated measurement [14]. Health, as a construct for people experiencing homelessness, has been overlooked in a hard-to-reach group who do not easily engage with traditional services until there is a crisis. The identification of valid and reliable tools is the first step towards developing proactive health assessments that will be relevant and acceptable to people experiencing homelessness.

This overall aim of this review was to identify health screening and assessment tools that have been developed for, or used with adults experiencing homelessness. The review:

- critiqued papers for methodological quality
- determined which health screening and assessment tools were validated or specifically developed for adults experiencing homelessness, and
- described the psychometric properties and utility of those tools.

## Methods

The review proposal was registered with PROSPERO (CRD42017068769). The conduct and reporting of the review findings follow the PRISMA guidelines [15].

### Search strategy

A preliminary database search identified common key words and MeSH terms that were then applied to the main search. The keywords included: homeless, homelessness, homeless persons, vagrancy, health status, health, health issues, health assessment and health screening. Where required, truncations (*) and MeSH terms were used in individual databases.

Library databases were searched in May 2017 and updated in September 2018. They included PubMed (and Medline), PsychInfo, Scopus, CINAHL and ERIC. The search was restricted to human studies published in English, and was not limited by date or type of article. Potentially-relevant articles were also identified through hand-searching of references cited in the reviewed articles. These were then screened according to the review method. The search strategy is provided in Additional file 1.

### Review method

Articles identified through the library database search were exported into EndNote v7 [16]. Duplicate articles were removed, and remaining articles were independently screened by title and abstract (AB and TD). Potentially-relevant articles were then screened in full text. Comparisons were made between reviewers at each screening phase and a third reviewer (SJG) resolved discrepancies.

### Exclusion criteria

This review did not include research about children and adolescents (younger than 18 years) experiencing homelessness, or studies that assessed health services for people experiencing homelessness, or studies that described tools that aimed to diagnose health conditions.

### Inclusion criteria

A two-step inclusion criteria was applied. Firstly, articles were included when they described the application of health screening or assessment tools for health issues related to adults experiencing homelessness (aged 18 years and over). Included articles also explicitly described the study sample as experiencing homelessness as per those proposed by the Australian Bureau of Statistics’ (ABS) definition of homelessness and provided in the introduction of this paper [5].

Secondly, the assessment and screening tools reported in these articles were aggregated to identify a sub-set, for which claims had been made about being developed for or with, and/or validated with people suffering homelessness. Where necessary, additional developmental literature for each tool was sourced for information on whether, and how the tool had been validated for the target population. This was completed by initially checking the reference lists and/or undertaking an independent literature search for additional material on the tool itself. Studies that used tools which had not been validated for the target population were noted, but no further analysis of methodological quality was undertaken.

### Data extraction

Data were extracted to detail all the articles by author, publication year, study population, setting, purpose, tool descriptions, condition/s assessed, primary outcomes, and tool psychometric properties (if applicable). Authors were contacted for further information when there was a lack of detail regarding the tool.

### Hierarchy of evidence

The hierarchy of evidence of the articles reporting on validated tools was assessed based on the National Health and Medical Research Council of Australia (NHMRC) recommendation [17]. The NHMRC hierarchy of evidence is internationally used in evidence reviews. It is based on the CEBM hierarchy model in which the hierarchy of evidence for diverse research questions contains different types of research designs (fit for purpose). It was appropriate for this review because of the likely heterogeneity of research designs.

### Critical appraisal

This took two forms.

1. Two reviewers independently appraised the methodological quality of the articles using validated tools relevant to the study design. These were 1) McMaster University Quantitative Critical Review Form [18], 2) McMaster University Qualitative Critical Review Form [19] or 3) the Leeds Evaluative Tool for Mixed Method Studies [20]. A third reviewer (SJG) arbitrated on discrepancies between reviewers, when required. Seven individuals conducted paired appraisals (AB, TD, SG, NB, MK, TB, CH). The McMaster critical appraisal instruments (designed for quantitative and qualitative designs) provided a ubiquitous approach to scoring methodological quality, without the need to use different tools for different designs (for instance the CASP suite). The McMaster critical appraisal tool provided a standardised way of comparing methodological quality across diverse research designs.
2. To assess the psychometric properties of the tools for which claims had been made regarding validation in the target population, the International Centre for Allied Health Evidence (iCAHE) Ready Reckoner was used. This is a structured checklist of psychometric properties and utility of application, developed for assessment and outcome measures [21]. The Ready Reckoner allows structured standardised comparison of psychometric properties of assessment instruments. It requires reviewers to identify the developmental literature on assessment instruments, and extract information on what psychometric properties were tested (and how), and what information is provided on utility. To gain a point for each category in the Ready Reckoner, instruments needed to have tested for, and reported on, the values for each psychometric test. The values themselves are not reported in the Reckoner, as the Reckoner is simply a way of summarising the amount and type of testing to which an instrument has been subjected during developmental phases. Moreover, as values are often dependent on sampling strategies, sample size and comparison instruments, it generally requires a great deal more information to interpret a value, thus reporting a value on its own can be misinterpreted. As completion of the Ready Reckoner requires access to the developmental references, these are cited, and readers interested in exploring more about an instrument can access further information efficiently to make their own judgements.

### Data synthesis

Tools were categorized to; 1) health screening or 2) health assessment.

## Results

### Search results

**Fig. 1:**
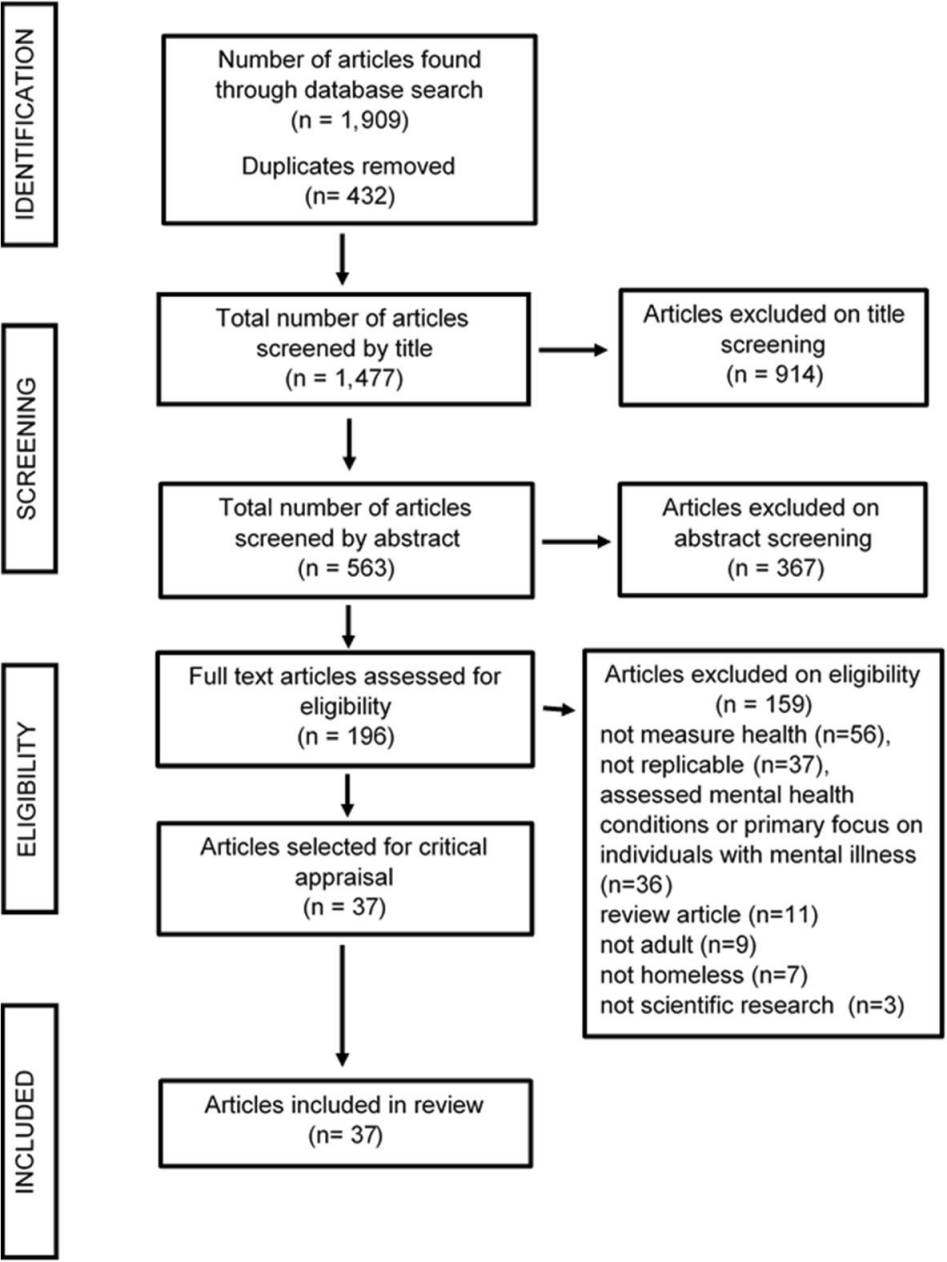
PRISMA flow diagram detailing the search and selection process

The database search and screening process is summarized in the PRISMA flow diagram (Fig. 1).

### Potentially-relevant studies and the tools they reported on

Of 2122 identified studies, 37 were identified as potentially-relevant (Table 1). These studies reported on 73 health screening or health assessment tools, across 11 domains: oral health [22–32]; health related quality of life, and health status [9, 33–48]; nutrition [49–56]; psychological and cognitive function [9, 22, 34, 35, 41, 42, 44, 56–59]; substance use [28, 33–36, 51, 57]; injury [29, 30, 36, 58]; chronic conditions [33, 44, 45, 60, 57]; demography and anthropometry [34–40, 44, 49, 51–55, 60]; functional decline and frailty [9, 37, 38, 54]; hearing and vision [9, 36, 61]; and pelvic floor health [9].

**Table 1:** Assessment instruments reported in the included literature, developmental reference(s) for the instrument (DRI) if relevant to homeless populations, and population(s) on which the instrument had been developed or validated. Shaded tools are validated or developed with people experiencing homelessness

Instrument and citation	DRI	Validation populations
Domain 1: Oral health		
WHO Oral health assessment guidelines [29, 30]		Adults and children worldwide Relevant to homeless, but not specifically validated
Decay (cavitation and visual dentine caries) and missing permanent teeth (D_3CV_MFT) [29, 30]		Children worldwide Relevant to homeless, but not specifically validated
Ellis standard classification [29]		Adults and children worldwide, relevant to homeless, but not specifically validated
Shortened Oral Health Impact Profile (OHIP-14 tool) (inc oral health-related quality of life interview) [22]		Adults 60+ years, relevant to homeless, but not validated
Community Periodontal Index (CPI) [23, 24, 29, 30]		Adults and children worldwide, relevant to homeless, but not specifically validated
Dental health measure [35]		No available information on instrument
Modified Dental Anxiety Scale (MDAS) [22, 23]		Adults, validated in many languages. Relevant to homeless, but not specifically validated
Oral hygiene level - the plaque index [32]		No available information on process or reference to test
The Adult Dental Health Survey (ADHS) [24]		Minimum information on process or reference to test. Information extracted from NHS website on the survey (geography, sex, age, ethnic group, household details, general and dental health, experience of dental services and treatments, dental examination for tooth and gum health.
The Geriatric Oral Health Assessment Index (GOHAI) [27]		Older adult populations internationally, relevant to homeless, but not specifically validated
Global Self-Rated Oral Health [27]		Adult populations internationally. Relevant to homeless, but not specifically validated
Domain 2: Quality of Life and health status		
The Medical Outcomes Study (MOS) 12 Item Short Form Survey (SF-12) [33, 39, 60] derived from MOS SF-36	[48, 62]	Validated on international adult populations including homeless
World Health Organisation’s Quality of Life – short version (WHOQOL-BREF) [41] derived from WHOQOL-100 [26, 43]	[43, 63]	Validated on international adult populations including homeless
Rural Homelessness Interview Research Instrument [35]	[35]	Developed for homeless rural women and children Midwestern United States of America
Nottingham Health Profile [44]	[64–66]	Validated on international populations including homeless
Delighted-Terrible Faces Scale (DTFS) [44]	[66]	Visual quality of life health outcome measure relevant to all ages. Reported to have been validated on homeless people in a PhD thesis
EQ-5D [40, 46]		Adult quality of life health outcome measure, relevant to, but not specifically validated on, homeless people
General health status tool [37]		Limited information available on process, or references
Global Self-Rated General Health [27]		Developed for adult populations, relevant to, but not specifically validated for, homeless
Life Fulfilment Scale (LFS) [44]	[66]	Developed for people with epilepsy, reported as validated for homeless populations in a PhD thesis
Quality of Life Scale [38]		Developed on a diverse adult population (incl ethnic minorities, older people, low income people) and validated on healthy adults, and adults with a range of health issues (incl Post Traumatic Stress syndrome). Relevant to, but not specifically validated for, homeless
RAND Current Health Scale [36]		Adolescents and adults internationally, relevant to, but not specifically validated on, homeless people
Rosenberg Self-Esteem Instrument [27]		Adolescents and adults internationally, relevant to, but not specifically validated on, homeless people
The New General Self-Efficacy Scale [38]		Adult self-efficacy measure, relevant to, but not specifically validated on, homeless people
Colorado Coalition for the Homeless (CCH) Consumer Outcome scale [34]	[34]	Outcome measure developed specifically for homeless
Veterans Research and Development (RAND) 12-Item Health Survey [27]		Adult quality of life health outcome measure developed from SF-36 for veterans, relevant to, but not specifically validated on, homeless people. Tested for people with depression, and alcohol addiction
Brief Instrumental Functioning Scale [10]	[69]	Validated on international adult populations including homeless
SF36 Italian version [40]		Validated in Italy with healthy groups, chronic obstructive pulmonary disease, myocardial infarction, migraine and dialysis populations (Apolone cref in Lav)
Health and Lifestyle Questionnaire [51]		Formulated for people experiencing homelessness, yes/no responses about appetite meal frequency, money spent on food no validation.
Domain 3: Substance Use		
Addiction Severity Index (ASI) [9, 33]	[67, 68]	Validated on international adult populations including homeless
Behavior and Symptom Identification Scale (BASIS-32) [34, 35]		Adult mental health outcome measure, not in the public domain, elevant to, but not validated on, homeless people
Cut Down, Annoyed, Guilty and Eye Opener (CAGE) alcoholism screening test [36, 57]		General screening tool relevant to adolescents and adults, relevant to homeless but not specifically validated
Drug Abuse Screening Test (DAST) [34, 35]		Adolescents and adults internationally, correlates with MAST, relevant to homeless but not specifically validated
Michigan Alcohol Screening Test (MAST) [34, 35]		Adolescents and adults internationally, validated for mental health concerns, sexual misdemeanours, alcohol abuse, relevant to homeless but not specifically validated
Domain 4: Injury		
Brain Injury Screening Questionnaire (BISQ) [58]	[70]	Child, adolescent and adult internationally, validated on homeless persons, individuals with HIV seeking vocational rehabilitation, youth in the juvenile justice system, public schoolchildren, substance users, intercollegiate athletes, and other community-based samples
Domain 5: Chronic Conditions		
American Thoracic Society guidelines for spirometry assessment [60]		Adult populations internationally. Relevant to homeless, but not specifically validated
Chronic pain Grade Questionnaire (7-item) [57]		Adult populations internationally. Relevant to homeless, but not specifically validated
Domain 6: Demography, anthropometry, risk factors		
Anthropometry [34, 36–39, 44, 46, 53–55, 60]		Adult populations internationally. Relevant to homeless, but not specifically validated
Biochemical analysis [49, 52, 54, 55]		Adult populations internationally. Relevant to homeless, but not specifically validated
Dartmouth improve your Medical Care [39]		Adult populations internationally. Relevant to homeless, but not specifically validated
RAND Serious Symptom Index [36]		Adult populations internationally. Relevant to homeless, but not specifically validated
RAND Minor Symptom Index [36]		Adult populations internationally. Relevant to homeless, but not specifically validated
Domain 7: Functional Decline and frailty		
10-Item modified Barthel Index [56]		Older adult populations (or younger adults with disability) internationally Relevant to homeless, but not specifically validated
Fried criteria [9]		Older adult populations internationally, relevant to homeless, but not specifically validated
Modified Katz Activities of Daily Living Scale [9]		Older adult populations internationally, Relevant to homeless, but not specifically validated
RAND Functional status [37]		Adult populations internationally. Relevant to homeless, but not specifically validated
Domain 8: Vision and hearing		
Snellen Chart [9]		Children, adolescents, adults internationally. Relevant to homeless, but not specifically validated
Retinal camera, ophthalmoscope, air puff tonometer [61]		Adult populations internationally, relevant to homeless, but not specifically validated
Domain 9: Mental health, psychological and cognitive function		
Boston Naming Test (BNT) [59]		Originally developed for individuals with aphasia or other language disturbance caused by stroke, Alzheimer’s disease, or other dementing disorder, now available for children. Relevant to homeless, but not specifically validated
Centre for Epidemiological Studies Depression Scale [22]		Children, adolescents and adults worldwide, relevant to homeless but not specifically validated
Color Trails test [59]		Children, adolescents and adults worldwide, relevant to homeless but not specifically validated
Controlled Oral Word Association Test (FAS) [59]		Adolescents and adults worldwide, relevant to homeless but not specifically validated
Geriatric Depression Scale (GDS-12R) [56]		Older adults, validated on healthy, medically ill and mild to moderately cognitively impaired older adults, relevant to homeless but not specifically validated
Grooved Pegboard Test [59]		Children, adolescents, adults, validated on many conditions, relevant to homeless but not specifically validated
Mini Mental State Examination (MMSE) [10]		Adults worldwide, relevant to homeless but not specifically validated
Neuropsychological Assessment Questionnaire [59]		Adults worldwide, relevant to homeless but not specifically validated
Repeatable Battery for Assessment of Neuropsychological Status (RBANS) [58]		Developed for adults with dementia, now validated on many conditions, and for children & adolescents, relevant to homeless but not specifically validated
Rey-Osterrieth Complex Figure Design (Rey-O) [41]		Children, adolescents, adults worldwide, tested for dementia, relevant to homeless but not specifically validated
Ruff Figural Fluency Test (RFFT) [59]		Adolescents and adults worldwide, relevant to homeless, but not specifically validated
Stroop Color Word test [59]		Children and adolescents
The Beck Depression Inventory-II, BDI-II [35, 45]	[71]	Adolescents and adults worldwide, validated for homeless men
The Elderly Cognitive Assessment Questionnaire (ECAQ) [56]		Adults worldwide, tested for older adults, relevant to homeless, but not specifically validated
Trail Making Test [59]		Adults worldwide, tested for older adults and those with dementia, relevant to homeless, but not specifically validated
Trail Making Test Part B (TMT-B) [10]		
Wechsler Adult Intelligence Scale, Third Edition (WAIS-III) [59]		Adults worldwide, tested for older adults, relevant to homeless, but not specifically validated
Wechsler Memory Scale, Third Edition (WMS-III) [59]		Adults worldwide, tested for older adults, relevant to homeless, but not specifically validated
Wisconsin Card Sorting Test (WCST) [59]		Adults worldwide Relevant to homeless, but not specifically validated
Domain 10: Nutrition		
24-h diet recall [50]		Adults and children worldwide, relevant to homeless, but not specifically validated
DETERMINE Your Nutritional Health Checklist [56]		Older adults worldwide, relevant to homeless, but not specifically validated
Food frequency questionnaire [50]		Adults and children worldwide, relevant to homeless, but not specifically validated
National Health and Nutrition Examination Survey [60]		Adult population internationally, relevant to homeless by not specifically validated
Domain 11: Pelvic Floor Health		
International Consultation on Incontinence Questionnaire [9]		Adult population internationally, relevant to homeless by not specifically validated

### Description of tools validated for people experiencing homelessness

Eleven assessment tools (reported in 13 articles) had been developed for, and/or validated in people experiencing homelessness. These tools addressed domains of health-related quality of life and health status, substance use, injury, mental health and psychological and cognitive function. They comprised:

- Addiction Severity Index (ASI) [33]
- Beck Depression Index (BDI II) [35, 45]
- Brain Injury Screening Questionnaire (BISQ) [58]
- Brief Instrumental Functioning Scale [9]
- Colorado Coalition for Homelessness Consumer Outcome Scale [34]
- Delighted-Terrible Faces Scale (DTFS) [40]
- Rural Homelessness Interview Schedule [35]
- Life Fulfilment Scale (LFS) [44]
- Nottingham Health Profile [44]
- Short Form Survey-12 (SF-12) and/or Short Form Survey-36 (SF-36) [39, 60, 41]
- World Health Organization Quality of Life 100 (WHOQoL 100) and/or World Health Organization Quality of Life BREF (WHOQoL-BREF) [26, 42, 43]

Fifty-five other assessment and screening tools were potentially-relevant to persons experiencing homelessness, but no evidence was found that validity had been established with this target population. Potential relevance was assessed in terms of generalizability or transferrable constructs between the population with which they had been developed and tested, and people experiencing homelessness. This was defensible for measures which are common to the general adult population, such as anthropometry and demography; oral health; continence; vision and hearing but not risk factors. Other potentially-relevant tools were found for conditions which could well be experienced by people who were homeless, including substance use, mental health issues, inadequate diet, poverty, injury and chronic illness. There were a further five tools for which little could be found in terms of psychometric properties or utility.

Table 1 lists the tools, the studies in which they were reported, the populations with which the tools had been developed or validated (where available), and references for additional literature providing evidence that the tool was valid for people experiencing homelessness. These studies are highlighted for ease of identification. The characteristics table for all included studies is provided in Additional file 2.

### Hierarchy of evidence

Of the 13 studies that used validated tools for the target population, three were classified as NHMRC III-1 [34, 35, 44] (prospective observational cohort studies). Two studies involved the development and validation of new assessment tools [34, 35] and the third [44] tested previously validated outcome measures in a pre-post study of the impact of case management for people experiencing homelessness. The remaining studies were classified as NHMRC III-2 as they were all cross-sectional studies reporting on surveys or measurements taken at a single time point.

### Methodological quality of included studies

Methodological quality of the included studies ranged from 57.1% [38] to 90.9% [9, 34, 39]. For the NHMRC III-1 studies the median quality score was 72.7% (25th % 64.9-75th % 81.8), whilst for the NHMRC III-2 studies the median quality score was 81.8% (25th % 72.7- 75th % 90.9). The individual quality appraisal items for each potentially-relevant article are provided in Additional file 3.

### Ready reckoner scores

The psychometric properties and utility of the validated tools are summarized in Table 2. As all tools had been specifically validated and developed for people experiencing homelessness, the question on relevance was deleted. The best performing tool for the target population was the WHOQoL 100 or WHOQoL BREF (scoring 100%), followed by BDI II, SF-12 and SF-36, and then the ASI (all scoring 94%). The Colorado Coalition for Homeless Consumer Outcomes scale ranked best of the purpose-built tools (82%), with the Rural Homelessness Interview Schedule (RHI) scoring poorest (56%).

**Table 2:** Ready Reckoner psychometric properties and utility

	Instrument & study refs (see key)	RHI [35]	SF12 & 36 [33, 39, 60]	WHOQol [26, 43]	NHP [44]	ASI [9, 33]	CCH [34]	BIFS [10]	BISQ [58]	BDI [35, 45]	DTFS [44]	LFS [44]
	Validation reference(s)	[35]	[48, 62]	[43, 63]	[64–66]	[67, 68]	[34]	[69]	[70]	[71]	[66]	[66]
Validity	face	√	√	√	√	√	√	√	√	√	√	√
	content	√	√	√	√	√	√	√	√	√	√	√
	construct		√	√	√	√	√	√	√	√		√
	comparison	√	√	√	√	√	√	√		√	√	√
	sensitivity		√	√	√	√	√	√	√	√	√	√
	factors		√	√		√	√	√		√		
Reliability	inter-tester	NA	NA	NA	NA	√	√	NA	√	NA	NA	NA
	intra-tester		√	√		√			√	√		√
	test-retest		√	√	√	√	√	√		√		√
	internal consistency		√	√	√	√	√	√	√	√	√	√
Utility	< 20 items	√	√		√			√			√	√
	Number of items	8	12	26	13	200	42	6	119+	21	1	20
	Manual scoring	√		√	√	√	√	√	√	√	√	√
	< 15 min admin time	NS	√	NS	√		√	√	√	√	√	√
	Estimated time (mns)	NS	10	NS	10	60	12	5	12	10	2	12
	Norms		√	√		√				√		
	Cut off scores		√	√						√		
	No cost	√		√	√	√	√	√	√	√	√	√
	No limitations on use	√		√		√	√	√	√	√	√	√
Total		9	15	16	12	16	14	13	13	15	10	13
Possible total		16	16	16	16	17	17	16	17	16	16	16
% total		56%	94%	100%	75%	94%	82%	81%	76%	94%	62%	76%

Key to instrument references in included literature

a. *RHI* Rural Homelessness Interview

b. *SF-12, SF-36* Medical Outcomes Study (MOS) (SF-12 derived from SF-36)

c. *WHOQOL_BREF derived from WHOQOL-100* World Health Organisation Quality of Life

d. *NHP* Nottingham Health Profile

e. *ASI* Addiction Severity Index (ASI)

f. *CCH* Colorado Coalition Homeless Consumer Outcome scale

g. *BIFS* Brief Instrumental Functioning Scale

h. *BISQ* Brain Injury Screening Questionnaire (BISQ)

i. *BDI II* Beck Depression Index II)

j. *DTFS* Delighted – Terrible Faces Scale

k. *LFS* Life Fulfilment Scale

## Discussion

This systematic review is the first that we know of, that has collated information on the validity of health screening and assessment tools used for people experiencing homelessness. Accurate information on health needs can only be obtained by the use of psychometrically-sound tools applicable to this population. While 73 health screening and assessment tools were reported as having been used to collect information on a range of issues in people experiencing homelessness, only 11 had published evidence of psychometric testing applicable to this target population. These tools captured information regarding quality of life, health status, substance use, injury, and psychological and cognitive function.

No validated tools were identified that assessed oral health, chronic conditions, anthropometry, demography, nutrition, continence, functional decline and frailty, or vision and hearing. However, assessments of physical constructs (such as oral health, anthropometry, vision and hearing) could be applied to homeless people on a presumption of validity, because the constructs would be measured clinically in the same manner as for people living in dwellings.

On the basis of the findings of this review, information reported on chronic health conditions, demography, functional decline and frailty, nutrition and pelvic floor health in people experiencing homelessness, may not be valid indicators of their health. For instance, it cannot be assumed that someone ‘living rough’ is a ‘community dweller’, which was a common description of the population for which the non-validated tools for people experiencing homelessness had been developed or tested [9] (See Table 1).

The iCAHE Ready Reckoner summarises common interpretations of validity and reliability as extracted from the developmental literature, as a way of demonstrating how well and thoroughly, a tool has been tested [21]. However, it is important to view testing in terms of the real world experienced by the target population, as findings may be constrained by the capacity of individuals to interpret and complete assessments. For example, issues concerning literacy and memory recall (due to substance abuse, illness or dementia) may affect acceptability of the tool and the quality of data collected with it. Thus, tool utility for the target population also needs to be considered in terms of its language and literacy levels, how long it takes to complete the items, and how many items the tool includes. Other aspects which assessors may need to consider are whether the tool is freely available and whether there are population norms for comparison.

Human health research must align with the ethical principles of beneficence, non-maleficence, health maximization, efficiency, and respect for autonomy, justice and proportionality [72]. People experiencing homelessness are a particularly vulnerable population, therefore assessment tools should enable efficient collection of representative data, relevant to the individual, and be delivered with minimum intrusion and maximum likelihood of doing good [1, 7, 72].

The health screening and assessment tools identified in this review contained 60 individual measures, of which 55 had available developmental material suggesting that with further research, the tool might be made relevant to people experiencing homelessness (See Table 1). Assessments of physical constructs (such as oral health, anthropometry, muscle strength, balance) appear to have been applied to the study population on a presumption of validity, because the constructs would be measured in the same manner as people living in permanent dwellings. The importance of oral health assessment of the target population was highlighted by the 11 different oral health tools identified in this review (of which nine had evidence of development and application). Whilst none had been validated with people experiencing homelessness, two warrant further development in this population; the Shortened Oral Health Impact Profile (OHIP-14 tool incorporating oral health-related quality of life interview) [22] and the Geriatric Oral Health Assessment Index (GOHAI) [27]. Both had been developed for efficient application with older people, needing minimum physical assessment time and including questions on oral health quality of life. Similarly, the validity of objective measures such as anthropometry, vision and hearing could be readily applied in homeless populations, as testing would be presumed to be similar for any adult. Heterogeneity of people experiencing homelessness supports the need for validated tools appropriate to specific subgroups, for example; different sex-age groups, or people with varying language and reading capabilities.

Without evidence of validity, self-report tools of most concern regarding applicability to homeless populations include those measuring health domains such as chronic conditions, functional decline and frailty, cognitive function, continence and nutrition. Not only could concerns be raised about the capacity and willingness of homeless people to complete written self-reports (because of literacy, vision or trust) but also question relevance to their circumstances. Recalling information over a set time period is a common element in most self-report tools, and this assumes that events of interest have occurred within that time period, and that an individual’s memory allows them to be accurately recalled and expressed. This is particularly problematic in dietary intake tools where the types of foods used to prompt memory may be inappropriate to circumstances of homelessness, and frequency and location of eating may be variable [50, 56, 60].

Two tools were identified which had been developed directly for, and with input from, homeless people [34, 35]. These tools had moderate to good study methodological quality, but poor to moderate tool property quality. The latter reflects the specific nature of the study population, and includes additional testing for reliability and external generalizability. Both tools captured information leading to homelessness, employment options, education, children, housing options, and access to benefits. These constructs had been identified during interviews with people experiencing homelessness, and were included in the fit-for-purpose tools, rather than existing tools that had been adapted for homeless populations. Consulting with homeless adults appears to be essential in designing sensitive, comprehensive, and multidimensional tools that capture the range of health concerns of this specific population group [14, 72, 73].

The WHO global healthy ageing initiative promotes good health for all adults, irrespective of country or circumstance [73, 74]. People ‘living rough’ have been shown to have symptoms of aging much earlier than the general population [4, 7, 9, 10]. Therefore, a lens might be placed on comprehensive test batteries developed for older people, to adapt them for people experiencing homelessness. Concerns in the elderly such as poor balance, falls, vision loss, incontinence, arthritis, chronic pain and poor skin health may also be relevant to younger homeless people [2, 10]. However, it will be necessary to engage homeless people in determine which criteria are important to them. Improving the quality and comprehensiveness of screening and assessment tools to quantify the prevalence and impact of such conditions currently associated with ageing, but which may also be found in homeless populations will provide important new information on the true impacts of homelessness. Additionally, this will provide an improved understanding of accelerated aging associated with poverty and sleeping rough.

### Limitations

This review was conducted as comprehensively as possible, using an in-depth search approach, that focused on English-language health screening and assessment tools developed for, used in, or potentially applicable to, homeless populations. It did not include articles about assessment of the specific diseases which may occur in people experiencing homelessness (for instance cardiac or respiratory diseases, mental illnesses or infectious diseases). The findings have not been differentiated by age group, sex, location or geography (which limits comparative insights into variability by sex, and among homeless populations globally). Future research might address these knowledge gaps to better establish health screens and assessments specific to different types of homelessness, and different types of people experiencing it [6, 7].

## Conclusions

The findings of this review confirm the complexities of assessing, accurately representing and addressing the potentially poor health of homeless populations. Few studies have reported on validated and reliable health screening and assessment tools, informed by input from people experiencing homelessness. Improving harm reduction strategies and early identification of health conditions in adults experiencing homelessness is vital, recursively flowing to and from empirical evidence, government policies and targeted services to address these significant and long-standing health inequities. That said, if research is asking, and by extension reporting on, the ‘wrong’ items and measures, there is significant potential for discord between what is espoused as needed, versus what should be triaged, to advance the health of homeless people. Based on the findings of the current review we recommend the development and consistent use of a suite of measures informed by and validated for people experiencing homelessness. This is a vital first step to accurately capturing, reporting and addressing the complex health needs of this vulnerable population.

Summary box for policy and practice A large number of assessment and screening tools for issues potentially related to the complex and varied health needs of people suffering homelessness was identified in this review. However, very few had been co-designed with people suffering homelessness, and many used language, wording, or situational descriptors that were not relevant to the target population.

There were no validated tools to assess issues such as oral health, chronic health conditions, anthropometry, demography, nutrition, continence, functional decline and frailty, vision or hearing.

There is an urgent need to develop consistent and comprehensive health assessment and screening tools specific to the needs and concerns of subgroups of people experiencing homelessness. This will ensure that valid data is available to inform health policies and healthcare initiatives that are likely to be effective.

## Additional files

Additional file 1: Search terms for the review (DOCX 13 kb)

Additional file 2: Characteristics of included studies (*n* = 39) (DOCX 64 kb)

Additional file 3: Critical appraisal summaries (DOCX 28 kb)

## Data Availability

The information accessed and included in this systematic review is available via the references provided.

## Abbreviations

ABS: Australian Bureau of Statistics
ADHS: Adult Dental Health Survey
ASI: Addiction Severity Index
BASIS-32: Behavior and Symptom Identification Scale
BDI II: Beck Depression Index
BIFS: Brief Instrumental Functioning Scale
BISQ: Brain Injury Screening Questionnaire
BNT: Boston Naming Test
CAGE: Cut Down, Annoyed, Guilty and Eye Opener
CCHCO: Colorado Coalition Homeless Consumer Outcome scale
CPI: Community Periodontal Index
D3CVMFT: Decay (cavitation and visual dentine caries) and missing permanent teeth
DAST: Drug Abuse Screening Test
DETERMINE: Nutritional Health Checklist
DRI: Developmental reference instrument
DTFS: Delighted-Terrible Faces Scale
ECAQ: Elderly Cognitive Assessment Questionnaire
EQ-5D: Euro Quality of Life Group five dimensions survey (mobility, self-care, usual activities, pain/discomfort, and anxiety/depression)
FAS: Controlled Oral Word Association Test
GDS-12R: Geriatric Depression Scale
GOHAI: Geriatric Oral Health Assessment Index
HIV: Human immunodeficiency virus
iCAHE: international Centre for Allied Health Evidence
LFS: Life Fulfilment Scale
MAST: Michigan Alcohol Screening Test
MDAS: Modified Dental Anxiety Scale
MMSE: Mini Mental State Examination
MOS: Medical Outcomes Study
NHMRC: National Health and Medical Research Council of Australia
NHP: Nottingham Health Profile
OHIP-14: Shortened Oral Health Impact Profile
RAND: Research ANd Development
RBANS: Repeatable Battery for Assessment of Neuropsychological Status
Rey-O: Rey-Osterrieth Complex Figure Design
RFFT: Ruff Figural Fluency Test
RHI: Rural Homelessness Interview
SF-12: Short Form Survey-12
SF-36: Short Form Survey-36
TMT-B: Trail Making Test Part B
USA: United States of America
WAIS-III: Wechsler Adult Intelligence Scale, Third Edition
WCST: Wisconsin Card Sorting Test
WHO: World Health Organization
WHOQoL 100: World Health Organization Quality of Life 100
WHOQoL-BREF: World Health Organization Quality of Life BREF
WMS-III: Wechsler Memory Scale, Third Edition
