# Supplementary material for: Health assessments and screening tools for adults experiencing homelessness: a systematic review
Source: BMC Public Health. 2019 Jul 24;19:994. doi: 10.1186/s12889-019-7234-y (PMC6657068; doi:10.1186/s12889-019-7234-y)
Supplement: Supplementary file 1 — Search terms for the review (DOCX 13 kb) [file 12889_2019_7234_MOESM1_ESM.docx]

**APPENDIX 1 – Search strategy**

**Medline**

1. Homeless Youth/ or Homeless Persons/ or homeless.mp.
2. vagrancy.mp. or Homeless Persons/
3. 1 or 2
4. Health Status/ or Health/
5. health issues.mp.
6. 4 or 5
7. "Quality of Life"/ or Health Status/ or health assessments.mp. or "Severity of Illness Index"/ or "Surveys and Questionnaires"/
8. Mass Screening/ or health screening.mp.
9. 7 or 8
10. 3 and 6 and 9
11. limit 10 to (abstracts and English language and humans)

**Number of articles found: 401**

**PubMed**

1. Search (homeless*) OR vagrancy Filters: Humans; English
2. Search ((health*) OR "health status") OR "health issues" Filters: Humans; English
3. Search ((("health assessment*") OR "health screening*") OR questionnaire*) OR survey* Filters: Humans; English
4. Search (((((homeless*) OR vagrancy) AND Humans[Mesh] AND English[lang])) AND ((((health*) OR "health status") OR "health issues") AND Humans[Mesh] AND English[lang])) AND ((((("health assessment*") OR "health screening*") OR questionnaire*) OR survey*) AND Humans[Mesh] AND English[lang]) Filters: Humans; English
5. Search (((((homeless*) OR vagrancy) AND Humans[Mesh] AND English[lang])) AND ((((health*) OR "health status") OR "health issues") AND Humans[Mesh] AND English[lang])) AND ((((("health assessment*") OR "health screening*") OR questionnaire*) OR survey*) AND Humans[Mesh] AND English[lang]) Filters: Humans; English; Field: Title/Abstract

**Number of articles found: 689**

**PsychInfo**

1. exp HOMELESS MENTALLY ILL/ or exp HOMELESS/ or homeless*.mp.
2. exp Homeless/ or vagrancy.mp.
3. 1 or 2
4. exp Mental Health/ or exp Health/ or exp "Quality of Life"/ or exp Aging/ or health status.mp.
5. health.mp. or exp HEALTH/
6. exp Health/ or "health issues".mp.
7. 4 or 5 or 6
8. exp Psychological Assessment/ or exp Health Screening/ or exp Measurement/ or "health assessment*".mp.
9. exp Health Screening/
10. 8 or 9
11. 3 and 7 and 10
12. limit 11 to (human and English language and abstracts)

**Number of articles found: 225**

**Scopus**

TITLE-ABS-KEY ( ( *homeless**  OR  *vagrancy* )  AND  ( *health**  OR  *"health status"*  OR  *"health issues"* )  AND  ( *"health assessment*"*  OR  *"health screening"*  OR  *questionnaire**  OR  *"health tool"* ) )  AND  ( LIMIT-TO ( DOCTYPE ,  *"ar"* ) )  AND  ( LIMIT-TO ( EXACTKEYWORD ,  *"Human"* ) )  AND  ( LIMIT-TO ( LANGUAGE ,  *"English"* ) )

**Number of articles found: 800**

**CINAHL**

1. AB homeless OR AB vagrancy
2. AB health* OR AB health status OR AB ( health issues or medical problems ) OR AB ( health issues or health problems )
3. AB ( health assessment and physical examination ) OR AB health assessment tools OR AB health screening tools OR AB health questionnaire
4. S1 AND S2 AND S3

**Number of articles found: 6**

**ERIC**

Ab(homeless people and vagrancy) AND ab(health* OR "health issue*" OR "health status") AND ab("health assessment*" OR "health screening" OR "health tool" OR "questionnaire*" )

**Number of articles found: 1**

**Total number of articles found: 2,122**

**Duplicates: 497**

**Final number: 1,625**
