# Supplementary material for: Health assessments and screening tools for adults experiencing homelessness: a systematic review
Source: BMC Public Health. 2019 Jul 24;19:994. doi: 10.1186/s12889-019-7234-y (PMC6657068; doi:10.1186/s12889-019-7234-y)
Supplement: Supplementary file 2 — Characteristics of included studies (n = 39) (DOCX 64 kb) [file 12889_2019_7234_MOESM2_ESM.docx]

**APPENDIX 2 –Characteristics of included studies (n=39)**

| **Study reference, setting and aims** | **Sample** | **Tool** | **Domain** | **Outcomes** | **Intervention** | **Key findings** |
| --- | --- | --- | --- | --- | --- | --- |
| **Domain: Cognition, communication and brain injury** | | | | | | |
| **Anderson et al. 2014 (55)**  To examine cognitive performance among a sample of men in a residential unit of an urban homeless shelter and to compare the cognitive function of the homeless men who screened negative for a history of traumatic brain injury to those who screened positive.  *Toronto, Canada.*  *Specifically* – men | *n* ***=*** 34 | **Study design:** Cross-sectional | | | - Recruited from a men’s homeless shelter - Conducted over 3 months - Data collection occurred in a long-term care unit for older men - Randomly selected - Initial demographic questionnaire - Followed by two questionnaires | - 12 participants had a positive traumatic brain injury (TBI) screen - Overall mean score for the RBANS was in the 11.4^th^ percentile, which is below the average level expected - The group with TBI performed slightly worse on the RBANS but only significantly for the attention domain (p = 0.026) - Screening for TBI and cognitive impairment is feasible with the homeless population - It was unexpected that there was no difference between the cognition domains for people with or without a TBI - Recommended using a screening tool which will consider the needs of the population to reduce fatigue |
|  |  | Brain Injury Screening Questionnaire (BISQ) | - Injury | - Presence of brain injury |  |  |
|  |  | Repeatable Battery for Assessment of Neuropsychological Status (RBANS) | - Mental health, psychological and cognitive function | - Neuro-psychological status |  |  |
| **Lo 2001 (56)**  To investigate the cognitive sequelae of multiple traumatic brain injuries in a homeless population.  *United States of America* | *n* ***=*** 128 | **Study design:** Cross-sectional | | | - Participants were recruited through the Neuropsychology Clinic at Harbor-UCLA Medical Centre - The neuropsychology battery of tools was administered to people who attended a clinic - Data collected over a period of 4 years - Fourth-year doctoral students in clinical psychology and post-doctoral fellows conducted the assessments - A licensed clinical neuropsychologist supervised each test | - Participants were aged 18 to 62 years - 35.9% female and 64.1% male - Results did not show that cognitive functioning across 8 cognitive domains changed with multiple traumatic brain injuries - 75.8% of the participants has suffered at least one TBI during their lives - Homeless shelters and services should screen for TBIs - For facilities with limited funding, time or expertise, the cognizant test could be used to assess cognition |
|  |  |  | Mental health, psychological and cognitive function | Neuro-psychological status |  |  |
|  |  | Boston Naming Test (BNT) | Mental health, psychological and cognitive function | Language ability |  |  |
|  |  | Color Trails test | Mental health, psychological and cognitive function | Attention and concentration |  |  |
|  |  | Controlled Oral Word Association Test (FAS) | Mental health, psychological and cognitive function | Language ability, to spontaneously generate words that begin with a certain letter |  |  |
|  |  | Grooved Pegboard Test | Mental health, psychological and cognitive function; dexterity | Psychomotor speed, fine motor control and rapid visuo-motor coordination |  |  |
|  |  | Rey-Osterrieth Complex Figure Design (Rey-O) | Mental health, psychological and cognitive function | New verbal learning abilities |  |  |
|  |  | Ruff Figural Fluency Test (RFFT) | Mental health, psychological and cognitive function | Executive functioning |  |  |
|  |  | Stroop Color Word test | Mental health, psychological and cognitive function | Selective attention and cognitive flexibility |  |  |
|  |  | Trail Making Test | Mental health, psychological and cognitive function | Attention, information processing speed and executive functioning |  |  |
|  |  | Wechsler Adult Intelligence Scale, Third Edition (WAIS-III) | Mental health, psychological and cognitive function | General intelligence |  |  |
|  |  | Wechsler Memory Scale, Third Edition (WMS-III) | Mental health, psychological and cognitive function | Verbal and visual memory |  |  |
|  |  | Wisconsin Card Sorting Test (WCST) | Mental health, psychological and cognitive function | Executive functioning, frontal lobe |  |  |

| **Domain: General health** | | | | | | | | |
| --- | --- | --- | --- | --- | --- | --- | --- | --- |
| **Study reference, setting and aims** | **Sample** | **Tool** | | **Domain** | **Outcomes** | | **Intervention** | **Key findings** |
| **Brown et al. 2012 (9)**  Determine prevalence of common geriatric syndromes in older homeless adults.  *Boston, United States of America*  *Specifically* – 50 to 69 years | *n* ***=*** 247 | **Study design:** Cross-sectional | | | | | - Recruited from emergency, transitional and day shelters - Systematic random sampling used - Data collected during a 40 minute interview - Data was compared to characteristics from 3 cohorts: the Maintenance of Balance, Independent Living, Intellect, and Zest in the Elderly (MOBILIZE) of Boston Study, the National Health and Nutrition Examination Survey (NHANES) and the national Health Interview Survey (NHIS) | - 30% of subjects reported difficulty completing >1 activity of daily living (ADL) - More than 50% had fallen in the past year - Mean MMSE score was 26.3 (standard deviation, 3.1) - Cognitive impairment in 24.3% participants (MMSE <24) - 16% met frailty criteria - 39.8% had major depression - After multivariate adjustment, homeless people had higher rates of ADL impairment - Geriatric syndromes higher in homeless persons |
|  |  | Mini Mental State Examination (MMSE) | | - Mental health, psychological and cognitive function | - Presence of mental health disorder | |  |  |
|  |  | Trail Making Test Part B (TMT-B) | | - Mental health, psychological and cognitive function | - Attention, information processing speed and executive functioning | |  |  |
|  |  | Modified Katz Activities of Daily Living Scale | | - Functional Decline (FD) and frailty | - Function and level of independence | |  |  |
|  |  | The Brief Instrumental Functioning scale | | - FD and frailty | - Level of independence | |  |  |
|  |  | Fried criteria | | - FD and frailty | - Frailty | |  |  |
|  |  | Patient Health Questionnaire | |  |  | |  |  |
|  |  | Snellen chart | | - Hearing and vision | - Visual impairment measured by best-corrected vision | |  |  |
|  |  | International Consultation on Incontinence Questionnaire | | - Pelvic floor health | - Incontinence | |  |  |
| **Chiu et al. 2009 (33)**  Examine association between immigrant status and current health.  *Toronto, Canada*  *Specifically* – Immigrants | *n* ***=*** 1189 | **Study design:** Cross-sectional | | | | | - Contacted every homeless shelter in Toronto for recruitment - 58 shelters agreed - Recruited over 12 months during 2004 to 2005 - Selected participants at random from bed or meal lists - Research team administered face to face survey | - Recent immigrants less likely to have chronic conditions, mental health problems or alcohol problems - Recent immigrants also more likely to have better SF-12 physical health score - Recent immigrants were more likely to have financial and housing reasons for being homeless - Recent immigrants less likely to report mental health conditions or addictions than non-recent immigrants or Canadian-born people - When findings were adjusted for age, sex, race/ethnicity, education and income, results showed recent immigrants had better health outcomes - Length of time since immigration is a big factor on their health – lifestyle behaviors are adopted from non-immigrants which effects their health negatively |
|  |  | Face to face structured interview | | - Chronic conditions | - Diabetes, anemia, hypertension, heart disease or stroke, liver problems (inc. hepatitis) arthritis, rheumatism or joint problems, cancer, walking problems, limb loss or other handicap, HIV/AIDS | |  |  |
|  |  | Addiction Severity Index (ASI) | | - Mental health, psychological and cognitive function - Substance use | - seven potential problem areas for substance abuse people | |  |  |
|  |  | The Medical Outcomes Study 12 Item Short Form Survey (SF-12) | | - Quality of life and health status | - Self-reported mental health score - Self-reported physical health score | |  |  |
| **Cook, Farrell and Perlman 2007 (34)**  1. To assess housing, employment, benefits, physical health, mental health, substance use of homeless persons.  2. To undergo psychometric testing of instrument.  *Colorado, United States of America* | *n* ***=*** 132 | **Study design:** Cross-sectional | | | | | - Participants from 5 difference service programs at Colorado Coalition for the Homeless (CCH) - Participants approached by primary researcher - Participants completed self-report questionnaires and also interviewed - Participants signed a release of information form to allow researchers to access their clinical records - Accessed information regarding housing status, employment, benefits received, physical and mental health and any documented substance use - Correlations calculated between self-report data and the assessed records - Research staff followed up with CCH clinician asking the staff member to complete the CCH survey | - Participants in this study were more likely to have a mental health diagnosis than the general homeless population - Interrater reliability averaged r=0.58 - With additional training higher interrater reliability could be achieved - 100% agreement on scale items for content validity - Clinicians reported that the instrument was clinically relevant and easy to use - Appropriate validity and reliability - Recommended for assessment or service planning with homeless individuals |
|  |  | The Colorado Coalition for the Homeless (CCH) Consumer Outcome Scales | - Demography, anthropometry, risk factors - Mental health, psychological and cognitive function   Substance use | | | Housing, employment , benefits, physical health, mental health and substance use |  |  |
|  |  | Michigan Alcoholism Screening Test (MAST) | Substance use | | | Severity of alcohol related problems |  |  |
|  |  | Drug Abuse Screening Test (DAST) | Substance use | | | Assesses drug use over 12 months |  |  |
|  |  | Behavior and Symptom Identification Scale (BASIS-32) | Substance use | | | Degree of difficulty with substance abuse, daily life and other factors |  |  |
| **Craft-Rosenberg, Powell and Culp 2000 (35)**  Describe health status and health resources available for rural women and children.  *Midwestern United States of America*  *Specifically* – women | *n* ***=*** 31 | The CCH Consumer Outcome Scales | | | | | - Demography, anthropometry, risk factors - Mental health, psychological and cognitive function - Substance use | - Housing, employment, benefits, physical health, mental health and substance use |
|  |  | Michigan Alcoholism Screening Test (MAST) | | - Substance use | - Severity of alcohol related problems | |  |  |
|  |  | Drug Abuse Screening Test (DAST) | | - Substance use | - Assesses drug use over 12 months | |  |  |
|  |  | Behavior and Symptom Identification Scale (BASIS-32) | | - Substance use | - Degree of difficulty with substance abuse, daily life and other factors | |  |  |
|  |  | Dental health measure | | - Oral health | - Overall dental health | |  |  |
| **Gelberg and Linn 1989 (36)**  Determine physical health of homeless depending on where they congregate.  *Los Angeles, United States of America* | *n* ***=*** 529 | **Study design:** Cross-sectional | | | | | - Recruited from 19 sites where homeless individuals congregated - Data collected by one primary investigator and 6 students - Interviews conducted for 50 minutes followed by a physical examination and blood test - Self-reported for the tools used | - Shelter users were less likely to have injured skin (p<0.01), have stomach ‘flu’ (p<0.01), low serum urea nitrogen levels + low albumin-globulin ratios. - 62% of participants were unaware they had high BP - 54% of daily alcohol drinkers did not report consuming alcohol - 82% who reported vision problems did not have impaired distance vision |
|  |  | RAND Serious Symptom Index | | - Demography, anthropometry, risk factors | - Symptoms more serious than the minor index | |  |  |
|  |  | RAND Minor Symptom Index | | - Demography, anthropometry, risk factors - Hearing and vision - Oral health | - Minor symptoms such as itching, trouble with vision, tooth ache, vomiting, diarrhea or backache | |  |  |
|  |  | CAGE alcoholism screening test | | - Substance use | - Alcohol use | |  |  |
|  |  | RAND Current Health Scale | | - Quality of life and health status | - Self-rated health status | |  |  |
|  |  | Interviews, physical examination and blood test | | - Demography, anthropometry, risk factors - Injury | - Blood pressure (BP) | |  |  |
| **Gelberg et al. 1990 (37)**  To compare health of homeless with domiciled adults.  *United States of America* | *n* ***=*** 464 | **Study design:** Cross-sectional | | | | | - Collected data from June to August, 1987 - Data collected by medical students - 30 minute structured interview followed by a physical examination - Interview on demographic information and homeless characteristics - Participants completed assessment tools | - Homeless had >1 grossly delayed tooth compared to non-homeless that had none. - Homeless were significantly more likely to report weight loss >4.5kg (p<0.009), pain in feet when walking (p<0.003), serious visual problems (p<0.004), seizures (p<0.002) or chronic obstructive pulmonary disease (p<0.01) - Physical examination results did not differ greatly between groups |
|  |  | 30 minute structured interview and physical examination | | - Demography, anthropometry, risk factors   Skin health | - Weight, height, body mass index, skin folds, arm circumference, waist circumference   Blood pressure | |  |  |
|  |  | Obvious decay experience (D_3CV_MFT) | | - Oral health | - Oral decay | |  |  |
|  |  | General health status tool | | - Quality of life and health status | - General health | |  |  |
|  |  | RAND Functional status | | - FD and frailty | - Self-rated functional status | |  |  |
| **Gregg and Bedard 2016 (38)**  To describe physical activity experiences and barriers to PA for homeless.  *Canada*  *Specifically* – men | *n* ***=*** 18 | **Study design:** Cross-sectional | | | | | - Recruited from a homeless shelter - Participants completed the questionnaires with a research assistant in the room to clarify questions - A semi-structured interview conducted from 15 to 45 minutes - Interview was recorded for transcription - 2 interviewers coded interviews to determine themes and subthemes | - Some moderate correlations between physiological and psychological variables - Participants with stronger intention to exercise walked faster - Outcome: participants had low scores in flexibility - Would benefit from exercise program - 5 themes resulted from interviews: healthy lifestyle factors, exercise information, benefits of exercise, barriers to exercise and exercise experiences - All identified nutrition as part of a healthy lifestyle |
|  |  | The New General Self-Efficacy Scale | | - Quality of life and health status | - Self-efficacy | |  |  |
|  |  | Quality of Life Scale | | - Quality of life and health status | - Quality of life | |  |  |
|  |  |  | | - FD and frailty | - Sit and reach measure to assess flexibility - Grip strength test to assess muscle strength | |  |  |
|  |  |  | | - Demography, anthropometry, risk factors | - Body Mass Index | |  |  |
| **Hwang et al. 2011 (59)**  To describe characteristics and treatments for chronic pain, barriers to pain management, concurrent medical conditions and substance users.  *Toronto, Canada* | *n* ***=*** 152 | **Study design:** Cross-sectional | | | | | - Homeless adults recruited between September 2007 and February 2008 - 17 shelters in Toronto - Randomly selected by bed number - Eligible for inclusion if they had pain for >3 months - Participants responses were recorded verbatim and categorized - Concurrent medical conditions assessed by self-report - CAGE administered to those who reported alcohol use in past 3 months | - On chronic pain scale, 7.5% grade I (low disability, low intensity), 32.2% grade II (low disability, high intensity), 23.3% Grade III (high disability, moderately limiting), 37.0% Grade IV (high disability, severely limiting). - Barriers to self-management were stress of shelter life, inability to afford medication, poor sleeping conditions - Prescription/over counter, street drugs and alcohol used to treat pain - Pain most commonly in back (52%), knees (28.9%), shoulders (21.2%) - Participants with more severe pain had greater barriers (p<0.001) |
|  |  | Interviewer led questionnaire | | - Chronic conditions - Pain | - Depression, arthritis, asthma, liver problems or hepatitis, hypertension, migraine, COPD, stomach or intestinal ulcers, heart disease, other conditions - medications and other therapies | |  |  |
|  |  | Chronic Pain Grade Questionnaire (7-item) | | - Chronic conditions | - Pain intensity, disability, management, control and duration in past 6 months | |  |  |
|  |  | CAGE alcoholism screening test | | - Substance use | - Alcohol use in past three months to assess dependency | |  |  |
| **Larson 2002 (39)**  Evaluate validity of SF-12 among users of homeless day shelter to assess health status.  *United States of America* | *n* ***=*** 145 | **Study design:** Cross-sectional | | | | | - Verbal administration of 2 questionnaires by trained interviewers to homeless adults - Conducted over 4 weeks   Comparison group comprised of residents in the same county; survey administered over the phone | - Trouble sleeping, dizziness, headaches and backaches were experienced by more than 50% of the homeless - 57% had one or more medical conditions - Homeless had significantly lower SF-12 scores than the general population, except for those earning less than $15,000 per year - Summary scores and subscales yielded satisfactory convergent validity coefficients ranging from 0.62 to 0.88   Internal consistency (Cronbach alphas 0.82 for physical health and 0.79 for mental health) |
|  |  |  | | | | |  |  |
|  |  | Dartmouth Improve your Medical Care survey | Demography, anthropometry, risk factors | | | Functional health, clinical symptoms, medical conditions and health risk |  |  |
|  |  | Short Form 12 item survey (SF-12) | Quality of life and health status | | | Health status |  |  |
| **Leverato, Bocci, Troiano, Messina, Nante 2017 (40)**  Health status of people living in a municipal dorm  *Padua, Italy* | *n* ***=*** 73 | **Study design:** Cross-sectional | | | | | Compared homeless outcomes to general Italian population | Homeless had lower health status, poorer mental health, higher smoking rates, greater alcohol intake, greater presentations to emergency department, more chronic health conditions and did not participate in health screening as much as general population |
|  |  | Questionnaire | Sociodemographic and medical history | | | Gender, age, nationality, qualification, height, weight, time spent at dorm, period of homelessness, alcohol, smoking, availability of GP, emergency presentations, hospital admissions, medical conditions, participation in health screenings |  |  |
|  |  | Short Form -36 | General health 8 scaled scores, the lower the score the greater the disability | | | Vitality, physical functioning, bodily pain, general health perceptions, physical role functioning,  emotional role functioning,  social role functioning,  mental health |  |  |
|  |  | EQ-5D | Health-related quality of life | | | Descriptive five dimensions: mobility, self-care, usual activities, pain/discomfort and anxiety/depression  Visual analogue scale. |  |  |
| **Usherwood and Jones 1993 (41)**  To measure perceived health and health service use.  *United Kingdom* | *n* ***=*** 104 | **Study design:** Cross-sectional | | | | | - Questionnaire developed for anonymous self-completion - Incorporated the SF-36 as well as sociodemographic information - Conducted on a single night in February 1992 | - 11% hospitalised in past 3 months, 23% out-patients and 15% visited ED - Poorer average perceived health compared to general population (p<0.001) - 74% at high risk of depressive illness - 4% at risk of dysthymia - Risk of major depression was associated with lower scores on general health, mental health, pain and social function dimensions of SF-36D |
|  |  | The Medical Outcomes Study Survey (SF-36) | Quality of life and health status | | | Quality of life |  |  |
|  |  | Questionnaire | Mental health, psychological and cognitive function | | | - Perceived health - Depression   dysthymia |  |  |

| **Domain: Oral health** | | | | | | |
| --- | --- | --- | --- | --- | --- | --- |
| **Study reference, setting and aims** | **Sample** | **Tool** | **Domain** | **Outcomes** | **Intervention** | **Key findings** |
| **Coles et al. 2011 (22)**  To determine the effect of dental health status, dental anxiety and oral-health-related quality of life upon homeless people’s experience of depression.  *Scotland* | *n* ***=*** 598 | **Study design:** Cross-sectional | | | - Training day for the participating dentists, dental nurses, dental hygienists, oral health promoters, and public health nurses for homeless people   - Overview of the questionnaire   - Standardization of the clinical oral health data collection   - Scottish National Dental Inspection Program - Dental health team visited the locations and participants self-completed the questionnaires | - 76% had previously accessed dental care due to toothache or problems with their teeth, gums or dentures - Mean number of decayed teeth was 4.68 - Mean number of extracted teeth was 7.58 - Mean number of restored teeth was 3.63 - Mean number of teeth affected by obvious decay was 15.87 - 20% of the participants were dentally phobic - 26% reported feeling self-conscious very often about the appearance of their teeth - 12% stated that their lives were less satisfying because of problems with their mouth and teeth - 57% of participants were suffering from a mental illness - The relationship between dental anxiety, oral health related quality of life and depression showed that decayed and missing teeth predicted depression through indirect pathways |
|  |  | Modified Dental Anxiety Scale (MDAS) | - Oral health | - Dental anxiety |  |  |
|  |  | Shortened Oral Health Impact Profile (OHIP-14) | - Oral health | - Oral health status |  |  |
|  |  | Centre for Epidemiological Studies Depression Scale | - Mental health, psychological and cognitive function | - Depression |  |  |
|  |  | Oral health examination conducted using the World Health Organization guidelines | - Oral health | - Oral health diseases and conditions |  |  |
| **Collins and Freeman 2007 (23)**  To assess the oral health needs of homeless people residing in Belfast to allow for recommendations for service delivery to be made.  *Belfast, Ireland*  *Specifically* – People in hostels | *n* ***=*** 317 | **Study design:** Cross-sectional | | | - Recruited from a variety of hostels for single homeless people - Recruited over 18 months - Completed a questionnaire made up of the oral health related tools - Completed the questionnaire prior to the oral examination - Oral examination conducted by a community dentist in a private room of the hostel | - All participants were unemployed - 97% had no obvious sign of extra-oral or intra-oral pathology - 16% had soft tissue lesions - Number of missing teeth ranged from 1 to 24 - Number of filled teeth ranged from 0 to 22 - Only 8% who had the oral examination had no obvious signs of gingival or periodontal disease - Mean score for dental anxiety was significantly greater than normative mean scores (p<0.05) - Mean score for oral health-related quality of life was 14.8 |
|  |  | Modified dental anxiety scale (MDAS) | - Oral health | - Dental anxiety |  |  |
|  |  | Shortened Oral Health Impact Profile (OHIP-14) | - Oral health | - Oral health status |  |  |
|  |  | Oral health examination conducted using the World Health Organization guidelines | - Oral health | - Oral health diseases and conditions |  |  |
|  |  | Obvious decay experience (D_3CV_MFT) | - Oral health | - Oral decay |  |  |
|  |  | Community Periodontal Index (CPI) | - Oral health | - Severity and degree of periodontal diseases |  |  |
| **Daly et al. 2010 (24)**  To describe the oral health status, oral health care needs and oral health-related quality of life in homeless people; to explore if there is a relationship between oral health status and oral health-related quality of life.  *London, United Kingdom*  *Specifically* – Persons using homeless facilities | *n* ***=*** 192 | **Study design:** Cross-sectional | | | - Recruited from 10 facilities attended by homeless people - Four hostels - Four day centres - Data collected through clinical examinations and interviews - Sociodemographic information collected - Interview made up of tools - Trained examiner conducted the clinical screenings - For the interviews, clinicians read out the questions and completed them when the participant could not do so themselves | - Mean DMFT score was 15.5 (±7.0)   - Mean DT was 4.2 (±5.2)   - Mean MT 6.8 (±6.0)   - Mean FT 4.6 (±4.8)   - Older people had higher DMFT score and more missing teeth (p<0.001, p<0.05) - Mean CPI score was 1.9 (±1.2) - 76% participants had restorative needs   - Active decay of 1 to 25 teeth - 80% needed oral hygiene measures - 65% reported aching of their mouth - 62% reported discomfort upon eating foods - 44% reported feeling handicapped by oral conditions - Mean number of teeth present was 25.2 (±6.1) - Most common QoL impacts was dimension of pain - People with natural teeth reported significantly less impacts than those with dentures (p<0.05) |
|  |  | Oral health-related quality of life interview (OHIP-14 tool) | - Oral health | - Oral health status |  |  |
|  |  | The Adult Dental Health Survey (ADHS) | - Oral health | - Overall dental health |  |  |
|  |  | Community periodontal index (CPI) | - Oral health | - Severity and degree of periodontal diseases |  |  |
|  |  | Oral health examination conducted using the World Health Organization guidelines | - Oral health - Quality of life and health status | - Oral health diseases and conditions |  |  |
| **Figueiredo, Hwang and Quinonez 2013 (25)**  To identify and quantify the oral health status and needs of an adult homeless population; to determine how the homeless perceive their oral health and dental experiences; to correlate the presence of oral disease with length of homelessness and unemployment.  *Toronto, Canada* | *n* ***=*** 191 | **Study design:** Cross-sectional | | | - Participants recruited from homeless shelters - Shelters and participants randomly selected - Shelters were visited during busiest hours to increase recruitment - Data collected through structured questionnaire administered face-to-face interview - clinical oral examination - One trained dental professional completed all of the examinations | - 70% of homeless reported having good overall health - 60% reported having some kind of medical condition - 40% reported having used drugs - 20% reported having no dental treatment needs - Only 3% had no dental treatment needs from examination - 88% required treatment from examination but only 35.1% reported self-need for treatment - Those who were homeless or unemployed for more than 1 year had significantly higher DMFT scores than those who were in the same situation for less than a year (p<0.01) |
|  |  | Oral health examination conducted using the World Health Organization guidelines | - Oral health | - Oral health diseases and conditions |  |  |
|  |  | Obvious decay experience (D_3CV_MFT) | - Oral health | - Oral decay |  |  |
| **Ford, Cramb and Farah 2014 (26)**  To describe adverse impacts of oral health problems and general quality of life for a homeless population.  *Brisbane, Australia* | *n* ***=*** 58 | **Study design:** Cross-sectional | | | - Recruited over a 15 month period during 2012-2013 - Recruited from a central Brisbane homeless service - Participants completed a self-report survey which contained oral health surveys - Assistance was provided by case manager if needed - All invited to attend a University dental clinic for a free assessment - Data collected was compared with the 2011 Australia Bureau of Statistics (ABS) Census and 2004-06 National Survey of Adult Oral Health | - Participants were significantly younger than the Brisbane population - Oral health impacts were significantly greater in the homeless than the Brisbane population - Prevalence, extent and severity of oral health impacts were at least 3 times greater in the homeless population - General quality of life was significantly poor across all 4 domains in the homeless - Median number of missing teeth was 6 - Median number of decayed teeth was 6, which is greater than the general population - Number of dental treatment types required for an individual was significantly associated with their oral health related quality of life (p=0.03) |
|  |  | Oral health-related quality of life interview (OHIP-14 tool) | Oral health | Oral health status |  |  |
|  |  | World Health Organization’s Quality of Life – short version (WHOQOL-BREF) | - Quality of life and health status | - Quality of life |  |  |
|  |  | Survey of Adult Oral Health | - Oral health | - Oral health status |  |  |
| **Gibson et al. 2008 (27)**  To assess the oral health needs of a national sample of homeless veterans and compare the dental needs of homeless veterans participating in rehabilitation programs with domiciled veterans in addiction programs.  *USA.*  *Specifically* – veterans | *n* ***=*** 281 | **Study design:** Cross-sectional | | | - Recruited from two veterans homeless rehabilitation programs - Compared homeless veterans between the two sites - Veterans completed a self-administered questionnaire prior to receiving dental care - First part of the questionnaire was sociodemographic information - Second part consisted of different tools | - 72.9% were smokers - 64.8% rated overall oral health as fair/poor - CWT participants had significantly better GOHAI scores (p<0.05) - CWT participants more likely to have history of diabetes, gastrointestinal reflux disease or schizophrenia - DOM patients had larger history of drug use - DOM patients saw a dentist more often - GOHAI improvement significantly related to having fewer teeth at baseline (p=0.048), a lower baseline GOHAI score (p=0.033) and improvement in self-reported oral health (p=0.007) - Significant improvement in veterans’ perceived oral health after receiving dental care |
|  |  | Global Self-Rated Oral Health | - Quality of life and health status | - General health |  |  |
|  |  | The Geriatric Oral Health Assessment Index (GOHAI) | - Oral health | - Oral health |  |  |
|  |  | Rosenberg Self-Esteem Instrument | - Quality of life and health status | - Self-esteem |  |  |
|  |  | Global Self-Rated General Health | - Quality of life and health status | - General health |  |  |
|  |  | Veterans RAND 12-Item Health Survey | - Quality of life and health status | - Quality of life and disease burden - Selim Comorbidity Index score |  |  |
| **Hill and Rimington, 2011 (28)**  To assess the oral health needs of homeless people in London, Cardiff, Glasgow and Birmingham in order to allow recommendations for service delivery.  *United Kingdom* | *n* ***=*** 27 | **Study design:** Qualitative | | | - Opportunistic sampling - All staff working in specialist units were sent letter of invitation to participate - Two questionnaires: one for homeless people, one for dental staff - Homeless people:   - Medical history   - Reasons for being homeless   - Substance abuse - Dental staff:   - Attitudes of other dental professionals working with homeless   - Treatments   - Patient related issues   - Future of homeless dentistry - Both questionnaires piloted - Assistance provided to complete questionnaire when needed - Open-ended questions were coded into themes - Framework developed from results | - 17 staff completed questionnaire - 9 dentists - 7 nurses - 1 therapist - 27 homeless people - People experiencing homelessness reported having poor dental health - Main reason for attending dental clinic was dental pain (94%) and missing teeth (41%) - 45% only attend the dentist when in pain - 95% owned a toothbrush and 91% had access to toothpaste - 59% brush their teeth twice daily - Staff provided thoughts on attitudes of other professionals, service arrangements, health, type of work undertaken with homeless and service use - Staff reported dental health was poor - This group of homeless had greater numbers of dental caries than the people in the Adult Dental Health Survey |
|  |  | Questionnaires designed for study – available in manuscript |  |  |  |  |
| **Luo and McGrath 2006 (29)** To assess the oral health status, self-reported dental problems and dental needs among the homeless population.  *Hong Kong, China* | *n* ***=*** 140 | **Study design:** Cross-sectional | | | - Contacted four major organizations for recruitment - Data collected in March 2005 - Conducted through clinical examination and face-to-face interview - Demographic questionnaire - 2 interviewers conducted the interview (15 minutes) - 2 trained examiners conducted the oral examination - Duplicate examinations conducted on 10% of subjects to assess inter-examiner agreement | - 89% rated their oral health as poor or very poor - 52% had dental pain in the past 12 months - 28% had less than 20 natural teeth - Number of natural teeth was associated with age (p<0.001), employment status (p=0.009), and duration of homelessness (p=0.025) - 90% had dental caries - 75% had one or more decayed teeth - 96% had periodontal pockets - 15% had evidence of traumatized teeth |
|  |  | Oral health examination conducted using the World Health Organization guidelines | - Oral health | - Oral health diseases and conditions |  |  |
|  |  | Obvious decay experience (D_3CV_MFT) | - Oral health | - Oral decay |  |  |
|  |  | Community periodontal index (CPI) | - Oral health | - Severity and degree of periodontal diseases |  |  |
|  |  | Ellis standard classification | - Oral health | - Injury to teeth |  |  |
| **Luo and McGrath 2008 (30)**  To determine oral health status and its impact on the quality of life of homeless people and to identify factors associated with oral health related quality of life in this group.  *Hong Kong, China* | *n* ***=*** 147 | **Study design:** Cross-sectional | | | - Contacted four major organizations for recruitment - Data collected in March 2005 - Recruited people from centers, shelters and on the street - Conducted through clinical examination and face-to-face interview - Conducted by 2 trained examiners and interviewers - Completed all survey tools - Questionnaire was administered face-to-face - Took approximately 15 minutes | - 85% had a toothbrush - 47% brushed their teeth once a day - 51% had dental pain in the past 12 months - 70% reported they needed to see a dentist - 46% reported their last dental visit was more than 3 years ago - 92% had some form of missing, decayed or filled teeth - 79% had one or more decayed teeth - 90% rated their oral health as less than good - 88% said that their oral health impacted their daily life - Unemployed homeless reported more oral health burden than those who were employed (p<0.05) - Those who did not have a tooth brush had greater oral health burden (p<0.01) - Those with higher D_3CV_MFT scores experience more oral health burden on life quality (p<0.05) |
|  |  | Oral health examination conducted using the World Health Organization guidelines | - Oral health | - Oral health diseases and conditions |  |  |
|  |  | Obvious decay experience (D_3CV_MFT) | - Oral health | - Oral decay |  |  |
|  |  | Community periodontal index (CPI) | - Oral health | - Severity and degree of periodontal diseases |  |  |
|  |  | Ellis standard classification | - Injury | - Injury to teeth |  |  |
| **Richards and Keauffling, 2009 (31)**  To quantify the way that oral diseases affect the lives of homeless and vulnerable people.  *Wales* | *n* ***=*** 100 | **Study design:** Cross-sectional | | | - Included homeless and vulnerable adults using the services of the Cyrenians Community Centre, Swansea - Recruited over 3 months from February to May 2007 - First part of questionnaire was sociodemographic information - Second part was the tool listed - Staff assisted participants in completing their surveys if needed | - Mean number of teeth was 19.7 - 58 participants reported they brush their teeth daily - Mean score for the OHIP-14 was 21.8 - 38 experienced ten or more impacts at least occasionally and 11 experienced them very often - Most common impacts reported were toothache, discomfort, ability to relax, and feeling ashamed regarding the appearance of their teeth - Pain was the most common - There were more rough sleepers in the group with high-impacts on their teeth (p=0.004) - Results show that homeless people have increased prevalence of oral health impacts |
|  |  | Oral health-related quality of life interview (OHIP-14 tool) | - Oral health | - Oral health status |  |  |
| **Sfeatcu et al. 2011 (32)**  To assess the oral-systemic health and the treatment needs among institutionalised people in a homeless centre.  *Romania*  *Specifically* – living in a shelter | *n* ***=*** 51 | **Study design:** Cross-sectional | | | - Recruited from a homeless shelter - Entrants undergo compulsory medical examinations - Dental examinations were conducted first - Followed by a questionnaire | - D_3CV_MFT was 15.53 - 78.8% had missing teeth - 62.4% had decayed teeth - 29.4% were caries free - Oral hygiene level was poor - High levels of dental plaque - Score for Silness and Loe index was 2.3 |
|  |  | Oral health examination conducted using the World Health Organization guidelines | - Oral health | - Oral health diseases and conditions |  |  |
|  |  | Obvious decay experience (D_3CV_MFT) | - Oral health | - Oral decay |  |  |
|  |  | Oral hygiene level - the plaque index | - Oral health | - Oral hygiene |  |  |

| **Domain: Nutrition** | | | | | | | | |
| --- | --- | --- | --- | --- | --- | --- | --- | --- |
| **Study reference, setting and aims** | **Sample** | **Tool** | | **Domain** | **Outcomes** | | **Intervention** | **Key findings** |
| **Darnton-Hill and Truswell 1990 (47)**  To assess the thiamine status of homeless people.  *Sydney, Australia*  *Specifically* – men | *n* ***=*** 107 | **Study design:** Case control | | | | | - Recruited over a 2 year period from two hostels in inner Sydney - Thiamine assessed three ways:   - Dietary information collected   - 24-hour recall was taken   - Hostel menus were analyzed - Anthropometric measurements included: - Weight - Height - Upper arm circumference - Biochemical analysis performed extensively | - 43% ate three meals a day - 52% took vitamin supplements at least once a week - 77% of participants drank alcohol at least once a week - 68% drank 9 or more alcoholic drinks in a session - Average thiamine intake from 24-hour recall was 0.76 ± 0.49 mg per day (Australian recommendation is 1.1 mg per day) - More than 66% were consuming less thiamine than is recommended on the day of the study - People taking vitamin supplements were consuming significantly more thiamine than those who weren’t (p<0.0001) - Proportion of clinical signs which indicated thiamine deficiency were:   - Abnormal gait (30%)   - Abnormal reflexes (15-61%)   - Abnormal plantar responses (8%)   - Abnormal sensation of the lower limbs (37%)   - Prevalence of Wernicke-Korsakoff syndrome of 24% - 64% had normal thiamine status - 21% showed marginal thiamine pyrophosphate effect - 15% had deficient thiamine pyrophosphate effect |
|  |  | 24-hour recall | | - Oral health | - Overall dietary balance and quality | |  |  |
|  |  | Anthropometry | | - Demography, anthropometry, risk factors | - weight, height, body mass index, skin folds, arm circumference, waist circumference | |  |  |
|  |  | Biochemical analysis | | - Demography, anthropometry, risk factors | - blood biochemistry | |  |  |
| **Evans and Dowler 1999 (48)**  To mobilize food resources from the food retail industry to improve the nutrition intervention for nutritionally at-risk people in day centres and using soup runs.  *London, United Kingdom* | *n* ***=*** 423 | **Study design:** Case control | | | | | - Recruited from central London during a 7 month period - Survey conducted face-to-face - Day centers and soup runs approached - Participants were randomly selected - A center worker assessed the questionnaire prior to administration to determine if questions were appropriate - Pilot testing conducted - Each interview consisted of brief personal history followed by 24-hour diet recall and food frequency questionnaire - Participant reporting was compared to menu at center and soup run - Study provides details on the specific nutrient consumptions for men and women | - 420 people completed the food frequency questionnaire - Food group consumption less than once a week: - 20% consumed vegetables - 52% consumed fruit - 64% consumed salad - 63% consumed fruit juice - 62% consumed brown/whole meal products - Daily consumption of food groups:   - 25% consumed vegetables   - 13% consumed fruit or drank fruit juice   - 7% consumed salad   - 20% consumed brown/whole meal products - Strongest association with frequency of food group intakes was the subject’s accommodation status and their desire to alter their diet - People in youngest age group (15-24 years) consumed more fruit juice than other age groups - Middle age group (25-55 years) had highest consumption of brown/whole meal products - Men ate more vegetables than women (p=0.05) - Females drank more fruit juice than men |
|  |  | 24-hour diet recall | | - Nutrition | - Overall dietary balance and quality | |  |  |
|  |  | Food frequency questionnaire | | - Nutrition | - Overall dietary balance and quality | |  |  |
| **Fallaize et al. 2017 (49)**  Compare the nutritional status, dietary intake and mental wellbeing in a group of homeless with age and sex-matched housed individuals  *Reading, UK* | *n* = 150 | **Study design:** Cross-sectional | | | | | 1. Homeless people living rough (75) recruited from church drop-in center providing meals.  2. Initial stages of housing (75) recruited from hostels  Men and women  Two groups broadly matched on age, sex and ethnicity  Completion of questionnaires plus  1. Height  2. Weight  3. BMI using WHO classification  4. Mean 3 handgrip strength dominant hand  5. Blood pressure | Significantly more homeless people:  - Smoked  - Took illicit drugs  - Did not have enough to eat  - Did not have a good appetite  - Did not have cooking facilities available  - Only had 2 meals per day compared to 3 in the housed group  - Earnt less money on a week and had less money to spend on food  No significant difference in BMI, handgrip strength or systolic blood pressure.  Homeless people had:  - Significantly higher diastolic blood pressure  - Increased cardio-vascular risk using the QRISK-2  - Significantly higher somatic, depressive and anxiety symptoms  - Significantly higher mean daily intake total fat, salt and alcohol, lower carbohydrate, fibre, vitamin C, fruit, calcium, milk and protein intake  Recommend need for dietary intervention in the homeless community |
|  |  | European prospective investigation into cancer and nutrition (EPIC)-Norfolk FFQ | Nutrient intake | | | Questionnaire consisting of 130 food and drink items, frequency (9 options) in the last month |  |  |
|  |  | Patient health questionnaire: somatic anxiety and depressive symptoms (PHQ-SADS) | Symptoms of a mental health condition | | | Combines three questionnaire depression (PHQ-9), anxiety (GAD-7) and somatization (PHQ-15) |  |  |
|  |  | Health and lifestyle questionnaire (developed for this study and not provided in the paper) | Demography, smoking, alcohol, substance abuse, appetite, meal frequency, money spent on food | | | Yes/no responses |  |  |
| **Kubisova et al. 2008 (50)**  To define the prevalence of protein-energy malnutrition in a sample of Prague’s homeless population.  *Czech-Republic* | *n* ***=*** 201 | **Study design:** Cross-sectional | | | | | - Recruited from a Czech charity organization and the Bulovka University Hospital - Completed a demographic questionnaire - Provided fasting blood samples - Anthropometric measurements taken - CAGE completed at the end 3 | - 7% of women and 4% of men showed low blood protein - 57% of men and 56% of women showed one or more abnormalities in red blood cell counts   - Low erythrocytes   - Low hemoglobin   - Low hematocrit - Macrocytosis (in 18 men and 6 women) - 3% of men and 7% of women were underweight - 33% of men and 26% of women were overweight - 5% of men and 7% of women were obese - 5% of men had low body fat stores - 41% of participants reported losing more than 10% of weight over 6 months - 69% of women and 29% of men ate at charities daily |
|  |  | CAGE alcoholism screening test | | - Substance use | - Alcohol use in past three months to assess dependency | |  |  |
|  |  | Biochemical/blood analysis | | - Demography, anthropometry, risk factors | - erythrocytes - hemoglobin - hematocrit - blood protein | |  |  |
|  |  | Anthropometry | | - Demography, anthropometry, risk factors | - Height - Weight - Skin-fold measurements - Waist and arm circumference - Body mass index | |  |  |
| **Langnase and Muller 2001 (51)**  To investigate the possible associations between nutritional state, nutrition, addiction habits, and health in a homeless population in Hamburg, Germany.   1. *Germany* | *n* ***=*** 75 | **Study design:** Cross-sectional | | | | | - Conducted between May and June 1996 - Recruited from 2 centers for homeless people - Written questionnaire conducted containing socio-demographic information, food frequencies, nutritional habits and health status - Anthropometric data collected | - 53% were malnourished - 24% healthy weight - 23% obese - 13.3% had high blood pressure - 8% had high cholesterol - 15% had skin fold below the 25^th^ percentile - Diseases of the stomach (17.3%) and diseases of the liver (14.7%) were most common - People who were smokers had lower BMI (p<0.05) - Drug addicts were more malnourished - 76% of people had a normal dietary pattern - Fresh fruit and vegetables were food groups not consumed enough - Poor nutritional state was associated with drug abuse as well as prevalence of wasting disease |
|  |  | Anthropometry | | - Demography, anthropometry, risk factors | - Height - Weight - Body mass index - Triceps skinfold - Upper arm circumference | |  |  |
|  |  | Food frequency questionnaire | | - Nutrition | - dietary balance and quality | |  |  |
| **Luder et al. 1990 (52)**  To analyse dietary intake of homeless and marginally housed people and to evaluate the nutritional content of meals provided in drop-in centres.   1. *New York, United States of America* | *n* ***=*** 96 | **Study design:** Cross-sectional | | | | | - Surveys distributed over a week during February and every Thursday morning during November and December of 1987 - Program provides medical and social service care to homeless persons - Surveyed 2 daytime drop-in centers which provided 3 meals per day - Surveyed 2 long-term accommodations which provided 2 meals per weekday - Surveyed a single-room-occupancy hotel where occupants provided their own meals - 2 registered dietitians assessed nutritional intake using a 24-hour recall - Nutrient content of 24-hour recall calculated using computers - Results compared to the 1989 RDAs specific for age and sex - Menus and recipes obtained from shelters and nutritionally assessed | - 34% with mental illness - 39% with hypertension - 17% with heart disease - 16% with diabetes - 7% with tuberculosis - 7% with COPD - 5% with alcohol abuse - Participants exceeded the cholesterol intake recommendation by 30% - 56% had cholesterol intakes above the desired level - 7% of women and 5% of men were underweight - 61% of women and 47% of men were overweight or obese - 39% of homeless people were obese - Participants had inadequate dietary intake as the mean dietary adequacy score was 10.7 - The 2 drop-in centers provided well-balanced meals - Residents who ate there however had low dietary adequacy scores - The saturated fat and cholesterol content of homeless shelter meals should be improved - Modified diets should be available - Homeless people should be educated about choosing healthy foods |
|  |  | 24-hour recall | | - Nutrition | - Dietary balance and quality | |  |  |
|  |  | Biochemical/blood analysis and questionnaires | | - Demography, anthropometry, risk factors | - Body Mass Index | |  |  |
| **Magkos et al. 2004 (53)**  To assess the nutritional status of the homeless, to evaluate their dietary nutrient intake, and to identify groups at high risk of undernutrition following the Athens 1999 earthquake.  *Greece*  *Specifically* – left homeless after earth quake | *n* ***=*** 255 | **Study design:** Cross-sectional | | | | | - Survey conducted in Menidi, one of the more severely damaged suburbs of Athens - Data collected from 2 camps - Camp Aetos: coked food rations centrally distributed daily - Camp Kaputa: supplied only raw or canned foods weekly - Participants selected randomly by selected tent numbers - Anthropometric measures include:   - Weight   - Height   - Body mass index - 24-hour recall conducted by a trained interviewer - Dietary intake was evaluated against the United Kingdom Reference Values for Food Energy and Nutrients - Blood analysis conducted 2 | - Individuals from both camps had mean BMIs in the overweight category - Adults were consuming total kilojoule consumption that was lower than recommended - Inadequate for preserving good health in the long term - 70% of adults failed to meet energy requirements - 60% of adults failed to meet protein requirements - All hematological parameters fell within normal ranges |
|  |  | Anthropometry | | - Demography, anthropometry, risk factors | - Weight - Height - Body mass index | |  |  |
|  |  | 24-hour recall | | - Nutrition | - Dietary balance and quality | |  |  |
|  |  | Biochemical/blood analysis | | - Demography, anthropometry, risk factors | - Hematological parameters | |  |  |
| **Visvanathan R, Ahmad Z. 2006 (54)**  To identify factors associated with low BMI in older residents of shelter facility.  *Malaysia*  *Specifically* – Residents of shelter care facilities | *n* ***=*** 1081 | **Study design:** Cross-sectional | | | | | - 2 research assistants interviewed participants using questionnaires that were translated into Malaysian - Conducted between March and September 2003 - Research assistants were trained by one investigator - Sociodemographic information was collected followed by the tools listed | - 14% of participants were underweight, BMI <18.5 - Lack of family support was associated with lower BMI - Consumption of fruits, vegetables and milk were associated with good oral health, were less likely to have low BMI - Nutritional intake, social support and oral health play an important role in maintaining health weight |
|  |  | DETERMINE Your Nutritional Health Checklist | | - Nutrition | - Common risk factors for malnutrition in people over 80 | |  |  |
|  |  | Geriatric Depression Scale (GDS-12R) | | - Mental health, psychological and cognitive function |  | |  |  |
|  |  | The Elderly Cognitive Assessment Questionnaire (ECAQ) | | - Mental health, psychological and cognitive function | - Early detection of dementia | |  |  |
|  |  | 10-item modified Barthel Index | | - FD and frailty | - Independence of a patient | |  |  |

| **Domain: Quality of life** | | | | | | |
| --- | --- | --- | --- | --- | --- | --- |
| **Study reference, setting and aims** | **Sample** | **Tool** | **Domain** | **Outcomes** | **Intervention** | **Key findings** |
| **Garcia-Rea and LePage 2008 (42)**  Evaluate psychometric properties of the WHOQOL-100.  *Texas, United States of America*  *Specifically* – veterans | *n* ***=*** 250 | **Study design:** Cross-sectional | | | - Veterans from the Domiciliary Residential Rehabilitation and Treatment Program - WHOQOL-100 tool administered every 2 weeks during active rehabilitation program until discharge | - Adequate internal consistency for all domain and facet scores - Test-retest reliability varied (ICC of 0.56 to 0.86 - Intra class coefficient ranged from 0.71 to 0.85 - Limited information on rehab program - Includes classes to build life skills, job readiness, group therapy, cooking classes, addresses psychiatric and medical health |
|  |  | WHOQOL-100 | - Quality of life and health status | - Quality of life |  |  |
| **Garcia-Rea and LePage 2010 (43)**  Evaluate psychometric properties of the WHOQOL-BREF.  *Texas, United States of America*  *Specifically* – veterans | *n* ***=*** 389 | **Study design:** Cross-sectional | | | - Veterans recruited from the VA North Texas Health Care System’s 40-bed Domiciliary Residential Rehabilitation and Treatment Program - Homeless veterans completed the questionnaire | - Adequate internal consistency for all domains - Validity supported using the Personality Assessment Inventory |
|  |  | WHOQOL-BREF instrument | - Quality of life and health status | - Quality of life |  |  |
| **Graham‐Jones, Reilly and Gaulton 2004 (44)**  Assess effectiveness of a health advocated casework with homeless people.  *United Kingdom*  *Specifically* – over 16 years | *n* ***=*** 117 | **Study design:** Randomized Control Trial | | | - Client group from hostels or temporary accommodation - Participants allocated to alternating periods of 1 to 3 months to healthcare advocacy or usual care for 3 years - Control months, had usual care - Intervention months, saw health advocate - Baseline interview - Assessment tools self-completed - Participants completed questionnaires again after 3 months - Questionnaires distributed face-to-face or by post | - 45% had a chronic illness - People recruited to program early in their stay had greater improvements in health-related quality of life - Outreach group reduced emotional stress levels compared to control - Outreach group improved significantly in emotional stress (p<0.01) and sleep (p<0.05) - Health care advocate group improved significantly more on social isolation dimension - The health advocacy groups improved on all LFS change scores - Outreach advocacy group improved significantly compared to the control on the DTFS |
|  |  | Life Fulfilment Scale (LFS) | - Quality of life and health status | - Quality of life |  |  |
|  |  | Delighted-Terrible Faces Scale (DTFS) | - Quality of life and health status | - Life satisfaction |  |  |
|  |  | Nottingham Health Profile (NHP) | - Quality of life and health status - Mental health, psychological and cognitive function | - Distress and subjective health |  |  |
|  |  | Dartmouth Improve your Medical Care survey | - Demography, anthropometry, risk factors | - Functional health, clinical symptoms, medical conditions and health risk |  |  |
|  |  | Short Form 12 item survey (SF-12) | - Quality of life and health status | - Health status |  |  |
| **Sarajlija et al. 2014 (45)**  To assess health related QoL + depression in homeless persons to describe sociodemographic factors and health status.  *Belgrade, Serbia* | *n* ***=*** 104 | **Study design:** Cross-sectional | | | - Study conducted in January 2012 - Shelter for Adult and Elderly Persons - Questionnaires were translated in Serbian - Sociodemographic and health status questionnaire was designed for this study - All questionnaires were self-administered - Tools listed were completed - If participants could not self-administer the test they were excluded | - 35.6% participants had lifetime diagnosis of psychiatric disorder - Severe depression in 20.2% participants - Comorbidity of 3+ chronic diseases associated with severe depression (p<0.003)   - 1 chronic somatic disease present in 31.7%, >2 in 19.2% participants - 15.4% had chronic heart failure, 23.1% had chronic lung disease |
|  |  | The Beck Depression Inventory-II, BDI-II | - Mental health, psychological and cognitive function | - Depression |  |  |
| **Sun et al. 2012 (46)**  Compare health-related QoL among homeless with general population in Stockholm.  *Sweden*  *Specifically* – hostel residents over 16 years | *n* ***=*** 153 | **Study design:** Cross-sectional | | | - Face to face interviews - Interview conducted from 30 minutes to 6 hours - Postal survey to general population sample in 2006 - Survey was to be self-completed by general population | - Chronic illness 3 times more common in homeless people - Most problems for the homeless population were in the depression dimension - Most problems in the general population were in the pain/discomfort dimension - Degree of homelessness was related to health |
|  |  | EQ-5D to measure QoL | - Quality of life and health status | - Quality of life |  |  |
| **Usherwood and Jones 1993 (41)**  To measure perceived health and health service use.  *United Kingdom* | *n* ***=*** 104 | **Study design:** Cross-sectional | | | - Questionnaire developed for anonymous self-complete - Incorporated the SF-36 as well as sociodemographic information - Conducted on a single night in February 1992 | - 11% hospitalised in past 3 months, 23% out-patients and 15% visited ED - Poorer average perceived health compared to general population (p<0.001) - 74% at high risk of depressive illness - 4% at risk of dysthymia - Risk of major depression was associated with lower scores on general health, mental health, pain and social function dimensions of SF-36D |
|  |  |  |  |  |  |  |
|  |  |  |  |  |  |  |
| **Domain: Vision** | | | | | | |
| **Study reference, setting and aims** | **Sample** | **Tool** | **Domain** | **Outcomes** | **Intervention** | **Key findings** |
| **Shahid et al. 2012 (57)**  To provide ophthalmic remote health screenings for homeless populations to identify and refer vision-threatening disease.  *New Jersey, United States of America* | *n* ***=*** 341 | **Study design:** Case control | | | - Recruited participants from soup kitchens over a 10-month period - Screenings conducted over 4 hours - Screening team made up of 4-6 students, 1 imaging specialist and 1 on-site coordinator - All trained in ocular screening - Methodology explained extensively in manuscript - Ocular screening conducted along with questionnaire on sociodemographic information | - 105 participants (30.8%) had some type of vision-threatening disease - Glaucoma was most prevalent (32%) - 21% affected by cataracts - 5% diagnosed with diabetic retinopathy - Community screening can help to meet unmet healthcare needs and prevent blindness - Cost ~$12,750 USD per year |
|  |  | Examination | - Hearing and vision | - Extensive eye examinations; - use of a non-mydriatic retinal camera, - panoptic ophthalmoscope, automated air puff tonometer |  |  |

| **Domain: Chronic conditions** | | | | | | |
| --- | --- | --- | --- | --- | --- | --- |
| **Study reference, setting and aims** | **Sample** | **Tool** | **Domain** | **Outcomes** | **Intervention** | **Key findings** |
| **Snyder and Eisner 2004 (58)**  To determine the prevalence of obstructive lung diseases in homeless people.  *United States of America*  *Specifically* – >35 years | *n* ***=*** 68 | **Study design:** Cross-sectional | | | - Recruited homeless people from one shelter in October 2002 - Males were randomly selected - All females were included due to their small number - Trained interviewer conducted a face-to-face interview first - Sociodemographic information collected - After interview, primary investigator conducted the spirometry assessment - Followed strict protocol explained in manuscript | - Prevalence of obstructive lung diseases (OLD) was high - 29% reported cough - 21% reported chronic bronchitis - 33% reported dyspnoea on exertion - High prevalence of lung function impairment - 37% had a low FEV_1_ - Prevalence of obstructive lung diseases by spirometry was 15% - 60% with OLD did not report a physician diagnosis of an airway disease |
|  |  | The Medical Outcomes Study Survey (SF-36) | - Quality of life and health status | - Quality of Life |  |  |
|  |  | National Health and Nutrition Examination Survey | - Nutrition |  |  |  |
|  |  | American Thoracic Society guidelines for spirometry assessment | - Demography, anthropometry, risk factors - Chronic conditions | - cough - chronic bronchitis - dyspnoea of exertion - lung function impairment - FEV_1_ - obstructive lung diseases |  |  |
