# Supplementary material for: Health assessments and screening tools for adults experiencing homelessness: a systematic review
Source: BMC Public Health. 2019 Jul 24;19:994. doi: 10.1186/s12889-019-7234-y (PMC6657068; doi:10.1186/s12889-019-7234-y)
Supplement: Supplementary file 3 — Critical appraisal summaries (DOCX 28 kb) [file 12889_2019_7234_MOESM3_ESM.docx]

**Appendix 3 –** Critical appraisal summaries

**Key:** shaded references used validated assessment instruments

| **General health measures** | | | | | | | | | | | | | | | | | | | | | | | | | | | | | | | | | | | |
| --- | --- | --- | --- | --- | --- | --- | --- | --- | --- | --- | --- | --- | --- | --- | --- | --- | --- | --- | --- | --- | --- | --- | --- | --- | --- | --- | --- | --- | --- | --- | --- | --- | --- | --- | --- |
| **Study** | **SP** | | | | **L** | | | **Des** | | | | **Sample** | | | | | | **Outcomes** | | | | | | | **Intervention** | | | | | **Results** | | | | **C** | **out of 16** |
|  |  |  |  |  |  |  |  |  |  |  |  | **Det** | | | **Just** | | | **Rel** | | | | **Val** | | | **Det** | | | **CT** | **CI** | **Sig** | **A** | **ClI** | **DO** |  |  |
| Brown et al. (9) | ✓ | | | | 🗶 | | | **CS** | | | | ✓ | | | 🗶 | | | **Multiple tools** | | | | **Multiple tools** | | | ✓ | | | **NA** | **NA** | ✓ | ✓ | ✓ | **NA** | ✓ | 7 |
| Chiu et al. (33) | ✓ | | | | ✓ | | | **CS** | | | | ✓ | | | ✓ | | | ✓ | | | | ✓ | | | ✓ | | | **NA** | **NA** | ✓ | ✓ | ✓ | **NA** | ✓ | 10 |
| Cook et al. (34) | ✓ | | | | ✓ | | | **CS** | | | | ✓ | | | 🗶 | | | ✓ | | | | ✓ | | | ✓ | | | **NA** | **NA** | ✓ | ✓ | 🗶 | **NA** | ✓ | 9 |
| Craft-Rosenberg (35) | ✓ | | | | ✓ | | | **CS** | | | | ✓ | | | 🗶 | | | 🗶 | | | | 🗶 | | | 🗶 | | | **NA** | **NA** | NA | ✓ | 🗶 | ✓ | ✓ | 6 |
| Gelberg & Linn (36) | ✓ | | | | ✓ | | | **CS** | | | | ✓ | | | ✓ | | | 🗶 | | | | 🗶 | | | ✓ | | | **NA** | **NA** | ✓ | ✓ | ✓ | ✓ | ✓ | 10 |
| Gelberg et al (37) | ✓ | | | | ✓ | | | **CS** | | | | ✓ | | | **NA** | | | ✓ | | | | ✓ | | | **NA** | | | **NA** | **NA** | ✓ | ✓ | ✓ | **NA** | ✓ | 9 |
| Gregg, Bedard (38) | ✓ | | | | ✓ | | | CS | | | | ✓ | | | 🗶 | | | Multiple tools | | | | Multiple tools | | | ✓ | | | NA | NA | ✓ | ✓ | ✓ | NA | ✓ | 8 |
| Hwang et al (59) | ✓ | | | | ✓ | | | **CS** | | | | ✓ | | | ✓ | | | ✓ | | | | ✓ | | | ✓ | | | **NA** | **NA** | ✓ | ✓ | ✓ | **NA** | ✓ | 11 |
| Larson, (39) | ✓ | | | | ✓ | | | **CS** | | | | ✓ | | | 🗶 | | | ✓ | | | | ✓ | | | ✓ | | | **NA** | **NA** | ✓ | ✓ | ✓ | **NA** | ✓ | 10 |
| Levorato et al (40) | ✓ | | | | ✓ | | | **CS** | | | | ✓ | | | 🗶 | | | ✓ | | | | ✓ | | | ✓ | | | **NA** | **NA** | ✓ | ✓ | ✓ | **NA** | ✓ | 10 |
| Usher-wood & Jones (41) | ✓ | | | | 🗶 | | | **CS** | | | | ✓ | | | 🗶 | | | **✓** | | | | ✓ | | | 🗶 | | | **NA** | **NA** | ✓ | ✓ | ✓ | **NA** | ✓ | 6 |
| **Quality of life** | | | | | | | | | | | | | | | | | | | | | | | | | | | | | | | | | | | |
| **Study** | **SP** | | | | **L** | | | **Des** | | | | **Sample** | | | | | | **Outcomes** | | | | | | | **Intervention** | | | | | **Results** | | | | **C** | **out of 16** |
|  |  |  |  |  |  |  |  |  |  |  |  | **Det** | | | **Just** | | | **Rel** | | | | **Val** | | | **Det** | | | **CT** | **CI** | **Sig** | **A** | **ClI** | **DO** |  |  |
| Garcia-Rea et al (42) | ✓ | | | | ✓ | | | **CS** | | | | ✓ | | | 🗶 | | | ✓ | | | | ✓ | | | ✓ | | | **NA** | **NA** | ✓ | ✓ | ✓ | 🗶 | ✓ | 10 |
| Garcia-Rea et al (43) | ✓ | | | | ✓ | | | **CS** | | | | ✓ | | | ✓ | | | ✓ | | | | ✓ | | | ✓ | | | **NA** | **NA** | ✓ | ✓ | ✓ | **NA** | ✓ | 11 |
| Graham‐Jones (44) | ✓ | | | | ✓ | | | **RCT** | | | | ✓ | | | 🗶 | | | **Multiple tools** | | | | **Multiple tools** | | | ✓ | | | ✓ | 🗶 | ✓ | ✓ | 🗶 | ✓ | ✓ | 9 |
| Sarajlija et al. (45) | ✓ | | | | ✓ | | | **CS** | | | | ✓ | | | 🗶 | | | **Multiple tools** | | | | **Multiple tools** | | | ✓ | | | **NA** | **NA** | ✓ | ✓ | 🗶 | **NA** | ✓ | 7 |
| Sun et al. (46) | ✓ | | | | ✓ | | | **CS** | | | | ✓ | | | ✓ | | | ✓ | | | | ✓ | | | ✓ | | | **NA** | **NA** | ✓ | ✓ | ✓ | **NA** | 🗶 | 10 |
| **Brain injury or cognition** | | | | | | | | | | | | | | | | | | | | | | | | | | | | | | | | | | | |
| **Study** | | **SP** | | | | **L** | | | **Des** | | | | **Sample** | | | | | | **Outcomes** | | | | | | **Intervention** | | | | | **Results** | | | | **C** | **out of 16** |
|  |  |  |  |  |  |  |  |  |  |  |  |  | **Det** | | | **Just** | | | **Rel** | | | | **Val** | | **Det** | | | **CT** | **CI** | **Sig** | **A** | **CI** | **DO** |  |  |
| Anderson et al.(55) | | ✓ | | | | ✓ | | | **CS** | | | | ✓ | | | 🗶 | | | ✓ | | | | ✓ | | ✓ | | | **NA** | **NA** | ✓ | ✓ | 🗶 | **NA** | ✓ | 9 |
| Lo, (56) | | ✓ | | | | ✓ | | | R | | | | ✓ | | | ✓ | | | ✓ | | | | ✓ | | ✓ | | | NA | NA | ✓ | ✓ | ✓ | NA | ✓ | 11 |
| Solliday-McRoy et al. (60) | | ✓ | | | | ✓ | | | **CS** | | | | ✓ | | | 🗶 | | | ✓ | | | | ✓ | | ✓ | | | **NA** | **NA** | ✓ | ✓ | ✓ | **NA** | ✓ | 10 |
| **Oral health** | | | | | | | | | | | | | | | | | | | | | | | | | | | | | | | | | | | |
| **Study** | | | | **SP** | | | **L** | | | | **Des** | | | **Sample** | | | | | | | **Outcomes** | | | | | | **Intervention** | | | **Results** | | | | **C** | **out of 16** |
|  |  |  |  |  |  |  |  |  |  |  |  |  |  | **Det** | | | **Just** | | | | **Rel** | | | **Val** | | | **Det** | **CT** | **CI** | **Sig** | **A** | **ClI** | **DO** |  |  |
| Coles et al. (22) | | | | ✓ | | | 🗶 | | | | **CS** | | | ✓ | | | 🗶 | | | | ✓ | | | ✓ | | | ✓ | **NA** | **NA** | ✓ | ✓ | ✓ | **NA** | ✓ | 9 |
| Collins & Freeman (23) | | | | ✓ | | | ✓ | | | | **CS** | | | ✓ | | | 🗶 | | | | ✓ | | | ✓ | | | ✓ | **NA** | **NA** | ✓ | ✓ | **✓** | **NA** | ✓ | 7 |
| Daly et al. (24) | | | | ✓ | | | ✓ | | | | **CS** | | | ✓ | | | 🗶 | | | | ✓ | | | ✓ | | | ✓ | **NA** | **NA** | ✓ | ✓ | ✓ | **NA** | ✓ | 10 |
| Figueiredo et al (25) | | | | ✓ | | | ✓ | | | | **CS** | | | ✓ | | | ✓ | | | | ✓ | | | ✓ | | | ✓ | ✓ | ✓ | ✓ | ✓ | ✓ | 🗶 | ✓ | 11 |
| Ford, (24) | | | | ✓ | | | ✓ | | | | **CH** | | | ✓ | | | ✓ | | | | ✓ | | | ✓ | | | ✓ | **NA** | **NA** | ✓ | ✓ | ✓ | 🗶 | ✓ | 11 |
| Gibson et al. (27) | | | | ✓ | | | ✓ | | | | **CH** | | | ✓ | | | 🗶 | | | | 🗶 | | | 🗶 | | | 🗶 | **NAd** | **NAd** | ✓ | ✓ | ✓ | **NA** | ✓ | 7 |
| Hill and Riming-ton (28) | | | | ✓ | | | ✓ | | | | **CS** | | | ✓ | | | 🗶 | | | | 🗶 | | | ✓ | | | ✓ | **NA** | **NA** | 🗶 | ✓ | 🗶 | NA | ✓ | 7 |
| Luo and McGrath (29) | | | | ✓ | | | ✓ | | | | **CS** | | | ✓ | | | 🗶 | | | | ✓ | | | ✓ | | | ✓ | **NA** | **NA** | ✓ | ✓ | ✓ | NA | ✓ | 10 |
| Luo and McGrath (30) | | | | ✓ | | | ✓ | | | | **CS** | | | ✓ | | | 🗶 | | | | ✓ | | | ✓ | | | 🗶 | **NA** | **NA** | ✓ | ✓ | ✓ | NA | ✓ | 10 |
| Richards &Keauffling (31) | | | | ✓ | | | ✓ | | | | **CS** | | | ✓ | | | 🗶 | | | | ✓ | | | ✓ | | | ✓ | **NA** | **NA** | ✓ | ✓ | ✓ | **NA** | ✓ | 10 |
| Sfeatcu et al. (32) | | | | ✓ | | | ✓ | | | | **CS** | | | ✓ | | | 🗶 | | | | ✓ | | | ✓ | | | ✓ | **NA** | **NA** | 🗶 | ✓ | ✓ | **NA** | ✓ | 9 |
| **Nutrition** | | | | | | | | | | | | | | | | | | | | | | | | | | | | | | | | | | | |
| **Study** | | | **SP** | | | | **L** | | | | **Des** | | | **Sample** | | | | | | | **Outcomes** | | | | | | **Intervention** | | | **Results** | | | | **C** | **out of 16** |
|  |  |  |  |  |  |  |  |  |  |  |  |  |  | **Det** | | | **Just** | | | | **Rel** | | | **Val** | | | **Det** | **CT** | **CI** | **Sig** | **A** | **ClI** | **DO** |  |  |
| Darnton-Hill & Truswell (47) | | | 🗶 | | | | ✓ | | | | **CS** | | | 🗶 | | | 🗶 | | | | ✓ | | | ✓ | | | ✓ | **NA** | **NA** | ✓ | ✓ | ✓ | **NA** | ✓ | 8 |
| Evans & Dowler (48) | | | ✓ | | | | ✓ | | | | **CS** | | | ✓ | | | 🗶 | | | | ✓ | | | ✓ | | | ✓ | **NA** | **NA** | ✓ | ✓ | ✓ | **NA** | ✓ | 10 |
| Falliaze et al (49) | | | ✓ | | | | ✓ | | | | **CS** | | | ✓ | | | ✓ | | | | 🗶 | | | 🗶 | | | 🗶 | **NA** | **NA** | ✓ | ✓ | ✓ | NA | ✓ | 8 |
| Kubisova et al. (50) | | | ✓ | | | | ✓ | | | | **CS** | | | ✓ | | | 🗶 | | | | ✓ | | | ✓ | | | ✓ | **NA** | **NA** | 🗶 | ✓ | ✓ | **NA** | ✓ | 9 |
| Langnase & Muller (51) | | | ✓ | | | | ✓ | | | | **CS** | | | ✓ | | | 🗶 | | | | ✓ | | | ✓ | | | ✓ | **NA** | **NA** | ✓ | ✓ | ✓ | **NA** | ✓ | 10 |
| Luder et al. (52) | | | ✓ | | | | ✓ | | | | **CS** | | | ✓ | | | 🗶 | | | | ✓ | | | ✓ | | | ✓ | **NA** | **NA** | ✓ | ✓ | ✓ | **NA** | ✓ | 10 |
| Magkos et al (53) | | | ✓ | | | | ✓ | | | | **CS** | | | ✓ | | | 🗶 | | | | ✓ | | | ✓ | | | ✓ | **NA** | **NA** | 🗶 | ✓ | ✓ | ✓ | ✓ | 10 |
| Visvanathan & Ahmad  (54) | | | ✓ | | | | ✓ | | | | **CS** | | | 🗶 | | | 🗶 | | | | ✓ | | | ✓ | | | ✓ | **NA** | **NA** | ✓ | ✓ | ✓ | **NA** | ✓ | 9 |
| **Chronic health conditions** | | | | | | | | | | | | | | | | | | | | | | | | | | | | | | | | | | | |
| **Study** | | | **SP** | | | | **L** | | | **Des** | | | | **Sample** | | | | | | **Outcomes** | | | | | | **Intervention** | | | | **Results** | | | | **C** | **out of 16** |
|  |  |  |  |  |  |  |  |  |  |  |  |  |  | **Det** | | | **Just** | | | **Rel** | | | | **Val** | | **Det** | | **CT** | **CI** | **Sig** | **A** | **ClI** | **DO** |  |  |
| Shahid et al.(57) | | | ✓ | | | | ✓ | | | **CS** | | | | ✓ | | | 🗶 | | | ✓ | | | | ✓ | | ✓ | | **NA** | **NA** | 🗶 | ✓ | ✓ | **NA** | ✓ | 9 |
| Snyder (58) | | | ✓ | | | | ✓ | | | **CS** | | | | ✓ | | | 🗶 | | | ✓ | | | | ✓ | | ✓ | | **NA** | **NA** | ✓ | ✓ | ✓ | **NA** | ✓ | 10 |

Key: A = analysis, C = conclusion, CH = cohort study, CI = Co-intervention, ClI = clinical importance reported, CS = cross-sectional, CT = contamination, Det = detailed, DO = dropouts, Just = justified, L = literature, NA = not applicable, NAd = not addressed, R = retrospective data, RCT = randomized controlled trial, Rel = reliable, Sig = significance reported, SP = study purpose, Val = valid
